# MyD88-dependent inflammasome activation and autophagy inhibition contributes to *Ehrlichia*-induced liver injury and toxic shock

**DOI:** 10.1371/journal.ppat.1006644

**Published:** 2017-10-19

**Authors:** Muhamuda Kader, Mounia Alaoui-EL-Azher, Jennie Vorhauer, Bhushan B Kode, Jakob Z. Wells, Donna Stolz, George Michalopoulos, Alan Wells, Melanie Scott, Nahed Ismail

**Affiliations:** 1 Department of Pathology, University of Pittsburgh, Pittsburgh, Pennsylvania, United States of America; 2 Center for Biologic Imaging, University of Pittsburgh Medical School, Pittsburgh, Pennsylvania, United States of America; 3 Pittsburgh Liver Research Center, University of Pittsburgh, Pittsburgh, Pennsylvania, United States of America; 4 Department of Surgery, University of Pittsburgh, Pittsburgh, Pennsylvania, United States of America; 5 Department of Immunology, University of Pittsburgh, Pittsburgh, Pennsylvania, United States of America; DUMC, UNITED STATES

## Abstract

Severe hepatic inflammation is a common cause of acute liver injury following systemic infection with *Ehrlichia*, obligate Gram-negative intracellular bacteria that lack lipopolysaccharide (LPS). We have previously shown that type I IFN (IFN-I) and inflammasome activation are key host-pathogenic mediators that promote excessive inflammation and liver damage following fatal *Ehrlichia* infection. However, the underlying signals and mechanisms that regulate protective immunity and immunopathology during *Ehrlichia* infection are not well understood. To address this issue, we compared susceptibility to lethal *Ixodes ovatus Ehrlichia* (IOE) infection between wild type (WT) and MyD88-deficient (MyD88^-/-^) mice. We show here that MyD88^-/-^ mice exhibited decreased inflammasome activation, attenuated liver injury, and were more resistant to lethal infection than WT mice, despite suppressed protective immunity and increased bacterial burden in the liver. MyD88-dependent inflammasome activation was also dependent on activation of the metabolic checkpoint kinase mammalian target of rapamycin complex 1 (mTORC1), inhibition of autophagic flux, and defective mitophagy in macrophages. Blocking mTORC1 signaling in infected WT mice and primary macrophages enhanced bacterial replication and attenuated inflammasome activation, suggesting autophagy promotes bacterial replication while inhibiting inflammasome activation. Finally, our data suggest TLR9 and IFN-I are upstream signaling mechanisms triggering MyD88-mediated mTORC1 and inflammasome activation in macrophages following *Ehrlichia* infection. This study reveals that *Ehrlichia*-induced liver injury and toxic shock are mediated by MyD88-dependent inflammasome activation and autophagy inhibition.

## Author summary

Human monocytic ehrlichiosis (HME) is the most prevalent emerging infectious disease in the United States. *Ehrlichia chaffeensis*, etiologic agent of HME, is a Gram negative obligate intracellular bacterium transmitted by infected tick bites and can infect different cell type. Although *Ehrlichia* lack lipopolysaccharide (LPS), they induce potentially life threatening HME that mimic sepsis or toxic shock associated with multi-organ failure. The clinical diagnosis of HME is difficult, and definitive diagnosis is most often retrospective. Late antibiotic treatment is frequently ineffective in preventing disease progression to fatal multi-organ failure. Liver failure is one of the most serious complications and the most frequent cause of death in patients with HME, however we only have a limited understanding of how this liver failure is caused during fatal *Ehrlichia* infection. The objective of this study is to determine how LPS-negative *Ehrlichia* activates inflammatory responses in macrophages during *Ehrlichia* infection to promote liver damage. We show here that MyD88-signaling causes detrimental derangement of the immune system and subsequent liver damage by regulating two key innate immune events in macrophages: autophagy and inflammasome activation. Targeting host-pathogenic pathways in ehrlichiosis can be incorporated into future design of novel therapeutic approaches for HME.

## Introduction

Human monocytic ehrlichiosis (HME) is the most prevalent emerging tick-borne, life threatening rickettsial disease and is caused by the obligate intracellular bacterium *E*. *chaffeensis*. Patients with HME develop severe liver inflammation and dysfunction followed by multi-organ failure and toxic shock-like syndrome. Protective immunity against *Ehrlichia* is mediated by Th1 cells [1–4]. On the other hand, fatal ehrlichiosis in humans and mice is due to an excessive inflammatory response and induction of pathogenic innate and adaptive immune cells, including neutrophils, NK cells and CD8^+^ T cells causing immunopathology [5–7].

We recently showed that virulent *Ehrlichia* are sensed by cytosolic pattern recognition receptors (PRRs) such as the nucleotide binding domain leucine-rich repeat (NLR) inflammasome complexes, including NLRP3[8]. Other studies have shown activation of canonical inflammasome pathways by a variety of intracellular pathogen-associated molecular patterns (PAMPs) and damage-associated molecular patterns (DAMPs) lead to cleavage of pro-caspase-1 and secretion of IL-1β, IL-18 [9–13]. Recently, non-canonical inflammasome activation involving caspase-11 activation by LPS was identified [9, 11, 14–16]. Caspase-11 or caspase-1 activation can lead to production of IL-1β and IL-18, as well as promotion of pyroptosis, an inflammatory programmed cell death, and lytic release of IL-1α and HMGB1 [17–19].

Inflammasome activation is a double-edged sword, contributing to both protective anti-microbial responses as well as inflammation and cell death when excessively activated [18, 20–22]. Our previous studies indicated that inflammasome activation is pathogenic during fatal ehrlichial infection [7, 23–26]. IL-18 signaling mediated induction and expansion of pathogenic CD8^+^ T cells in murine model of fatal ehrlichiosis, which caused tissue damage [3, 23, 25]. Thus, regulation of inflammasome is critical for controlling *Ehrlichia* infection without causing collateral damage. Type-I interferon (IFN-I) is a major negative regulatory mechanism for inflammasome activation during infections with several intracellular bacteria [27–31]. Paradoxically, we and others have showed that type I IFN and IFN-I receptor (IFNαR) signaling positively regulates inflammasome activation during infection with *Ehrlichia*, *Francisella*, and *Streptococcus pneumoniae* [24, 30, 32–34]. Autophagy is another process that negatively regulates inflammasome activation. Autophagy involves the formation of double-membrane compartments (phagophores) that capture damaged host organelles and cytoplasm, as well as intracellular bacteria. Autophagic flux involves maturation of these phagophores into autophagosomes, which then fuse with lysosomes to form single-membrane autolysosomes where degradation of the autophagic cargo and subsequent recycling of proteins and ATP synthesis occurs [27, 35]. Induction of autophagy is mediated by several autophagy-promoting molecules including Atg5, Atg12, Atg16, Atg8/LC3 (LC3 is the mammalian homologue of yeast Atg8) and beclin1. Decreased production of any of these factors or decreased lipidation of Atg8/LC3 attenuate formation of autophagosomes and impairs autophagy process. While autophagy is a protective innate mechanism against facultative intracellular bacteria [27, 36], recent studies suggested that autophagy promotes survival and replication of obligate intracellular bacteria such as *Ehrlichia* and *Anaplasma*, as these bacteria capture nutrients through this process that promote bacterial growth and replication [37–39].

Unlike other Gram negative bacteria, *Ehrlichia* lack LPS [40–42], a major inducer of innate responses against these pathogens. The underlying mechanisms that regulate innate immune and inflammasome activation in macrophages during infection with LPS negative *Ehrlichia* remain elusive. Myeloid differentiation factor 88 (MyD88) is a major adaptor molecule downstream of several surface and cytosolic pattern recognition receptors [43]. In this study, we examined whether *Ehrlichia*-induced inflammasome activation in macrophages is regulated by MyD88 and the mechanisms involved in this regulation. Our data suggest dual protective and pathogenic roles of MyD88 in host response to *Ehrlichia*. MyD88 inhibited autophagy induction and controlled bacterial replication by activating mTORC1. On the other hand, MyD88-mediated mTORC1 activation induced host-pathogenic inflammasome activation and liver damage by blocking autophagosome-lysosomal fusion and mitophagy in macrophages.

## Results

### MyD88-signaling promotes *Ehrlichia*-induced immunopathology, and enhances protective immunity against *Ehrlichia*

**Fig 1:**
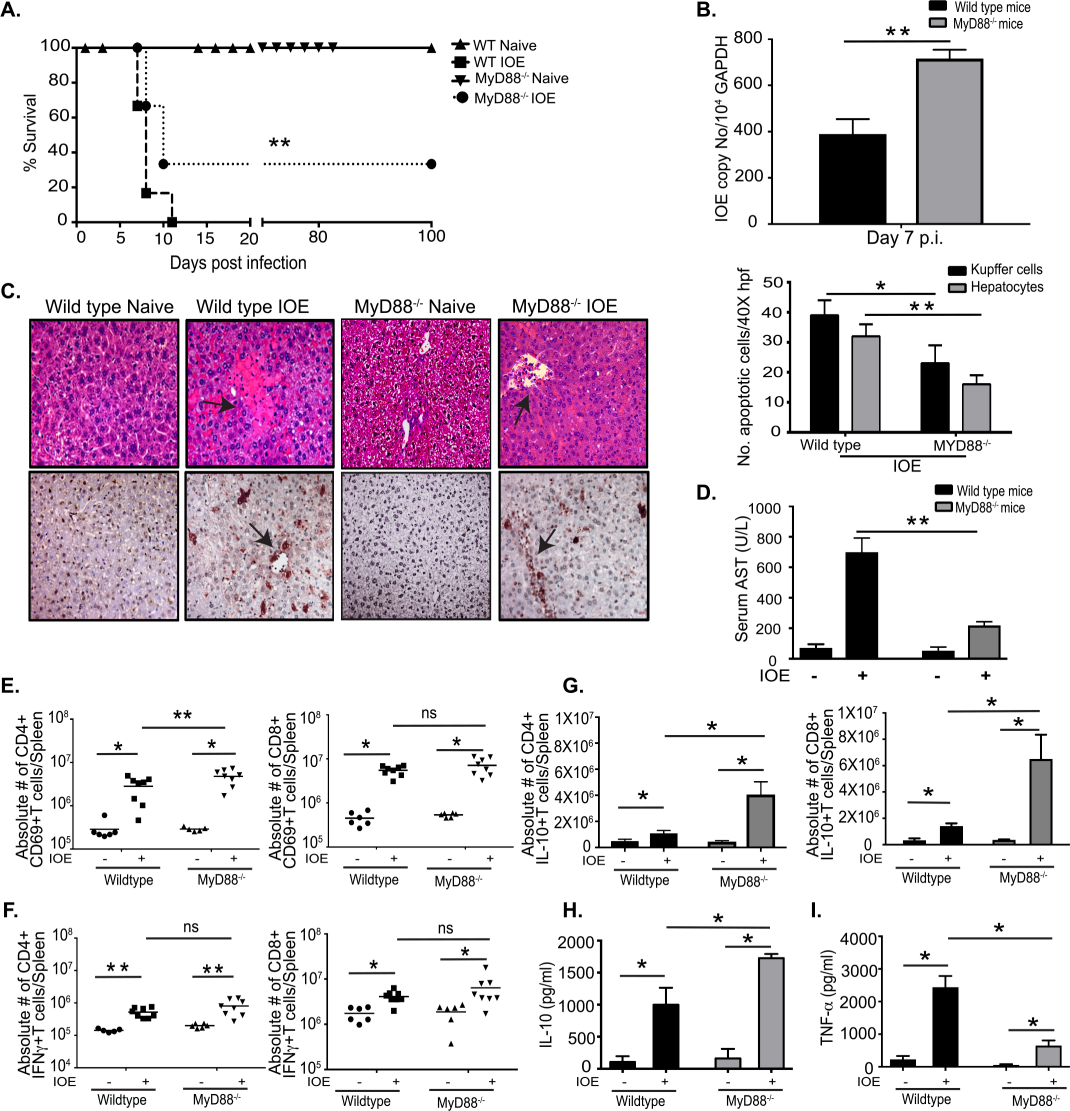
MyD88 signaling promotes host susceptibility to fatal *Ehrlichia*-induced toxic shock. (A) Survival of IOE-infected WT and MyD88^-/-^ mice, showing prolonged survival of MyD88^-/-^ mice compared to WT mice (n = 9/group). (B) Bacterial burden in liver of IOE infected WT and MyD88^-/-^ mice determined by quantitative real-time PCR on day 7 p.i. (C) H&E (upper) and TUNEL (lower) staining of liver sections showing the number of TUNEL positive Kupffer cells and hepatocytes per high power field (HPF) in uninfected and IOE-infected MyD88^-/-^ and WT mice on day 7 p.i. Arrows indicate example areas of necrosis (H&E) and TUNEL positive cells (TUNEL). Quantification of TUNEL positive Kupffer cells and Hepatocytes per HPF in WT and MyD88^-/-^ mice. (D) Levels of AST were detected in sera of uninfected and IOE infected WT and MyD88^-/-^ mice on day 7 p.i. Splenocytes were harvested from the indicated mice groups at day 7 p.i. and phenotypes of T cell subsets analyzed by flow cytometry following an *in vitro* stimulation with IOE antigens. (E) Absolute number of activated CD4^+^ and CD8^+^ T cells expressing CD69 in spleens at 7 days p.i. (F) Absolute number of CD4^+^ and CD8^+^ T cells producing IFNγ. (G) Absolute number of CD4^+^ and CD8^+^ T cells producing IL-10. Levels of IL-10 (H) and TNF-α (I) in sera from naïve and infected WT or MyD88^-/-^ mice measured by ELISA. Data shown in bar graphs indicate mean ±SD from three mice per group and are representative of three independent experiments. * P<0.05, ** P<0.01, ns = not significant.

To investigate the role of MyD88 in the pathogenesis of *Ehrlichia*-induced liver injury, we analyzed the host response to *Ehrlichia* in WT and MyD88^-/-^ mice infected with an ordinarily high lethal dose of IOE (10^4^ organisms/mouse). Consistent with our previous studies, 100% of IOE-infected WT mice succumbed to lethal infection by days 7–11 post-infection (p.i.). In contrast, only about 70% of MyD88^-/-^ mice succumbed to infection by that time point (**Fig 1A**). Interestingly, MyD88^-/-^ mice had a significantly higher bacterial burden in the liver on day 7 p.i. compared to WT mice (**Fig 1B).** H&E and TUNEL staining of the liver tissues from uninfected WT and MyD88^-/-^ mice exhibited similarly normal liver histology with no evidences of cell death or immune cell infiltration. However, liver tissue from IOE-infected WT mice exhibited evidence of severe liver damage on day 7 p.i. as indicated by multiple foci of hepatocyte necrosis and apoptosis as well as fatty changes. In contrast, IOE-infected MyD88^-/-^ mice had significantly attenuated liver injury at the same time point, as marked by significantly decreased TUNEL positive and necrotic (shown by H&E staining) hepatocytes and Kupffer cells, reduced magnitude of fatty changes/steatosis (a hallmark of ehrlichial hepatocyte infection)[3, 23, 24] (**Fig 1C**), as well as decreased serum level of aspartate transaminases (AST) levels when compared to infected WT mice (Fig 1D).

Type I response, mainly by CD4^+^ Th1 cells, is critical for protective immunity against *Ehrlichia*, while IL-10 production by T and non-T cells inhibits bacterial clearance [1, 5, 6]. To determine the potential mechanism responsible for impaired bacterial clearance in MyD88^-/-^ mice, we analyzed the phenotype of antigen (Ag)-specific T cells in the spleen of WT and MyD88^-/-^ mice on day 7 p.i. by flow cytometry (**S1 Fig**). There was no difference in the absolute number of CD4^+^ or CD8^+^ T cells between naïve WT and MyD88^-/-^ mice (**S1B, S1C and S1D Fig),** suggesting that MyD88^-/-^ mice do not have an altered immune phenotype at baseline that could influence their response to *Ehrlichia* infection. However, IOE infection of MyD88^-/-^ mice resulted in significantly higher percentage and number of activated Ag-specific CD4^+^ T cells expressing CD69 when compared to infected WT mice (**Fig 1E & S1B Fig),** although there was no significant difference in the activation of CD8^+^ T cells between the groups (**Fig 1E)**. IOE-infected WT and MyD88^-/-^ mice have significantly higher frequency of IFNγ^+^CD4^+^ Th1 cells and IFNγ^+^CD8^+^ Tc1 cells than corresponding naïve mice from each group, but there was no significant difference in the frequency of Th1 or type I CD8^+^ T cells between IOE-infected WT and MyD88^-/-^ mice **(Fig 1F and S1B Fig).**

We and others have shown that TNF-α and IL-10 are key mediators of protective immunity or immunosuppression during fatal IOE infection, respectively [6, 23, 25]. Thus, to further examine the potential mechanism that could account for impaired bacterial elimination in MyD88^-/-^ mice, we measured IL-10 and TNF-α production by CD4^+^ and CD8^+^ T cells at the single-cell level by flow cytometry **(S1C Fig),** and in culture supernatant by ELISA. Splenocytes from both naïve WT and MyD88^-/-^ mice have negligible numbers of Ag-specific TNF-α or IL-10 producing CD4^+^ and CD8^+^ T cells. However, IOE- infected MyD88^-/-^ mice have significantly increased Ag-specific IL-10^+^CD4^+^ T cells and IL-10^+^CD8^+^ T cells in their spleens compared to infected WT mice (**Fig 1G**). Although the frequency of Th1 cells did not differ significantly between infected WT and MyD88^-/-^ mice, the ratio of Ag-specific IL-10^+^: IFNγ^+^ producing CD4^+^T cells was significantly higher in infected MyD88^-/-^ mice compared to WT mice (**S1E Fig**). Additionally, sera from IOE-infected MyD88^-/-^ mice have significantly higher levels of IL-10 **(Fig 1H),** but lower levels of TNF-α (**Fig 1I**) as well as higher IL-10: TNF-α ratio when compared to WT mice **(S1F Fig**). The bias of adaptive immune responses in infected MyD88^-/-^ mice towards an immunosuppressive phenotype is consistent with higher bacterial burden in these mice. Together, these data suggest that while MyD88 mediates inflammatory liver injury, it also moderates the degree of *Ehrlichia*-induced immunosuppression and enhances bacterial elimination.

### Regulation of canonical inflammasome activation by MyD88 during *Ehrlichia* infection

**Fig 2:**
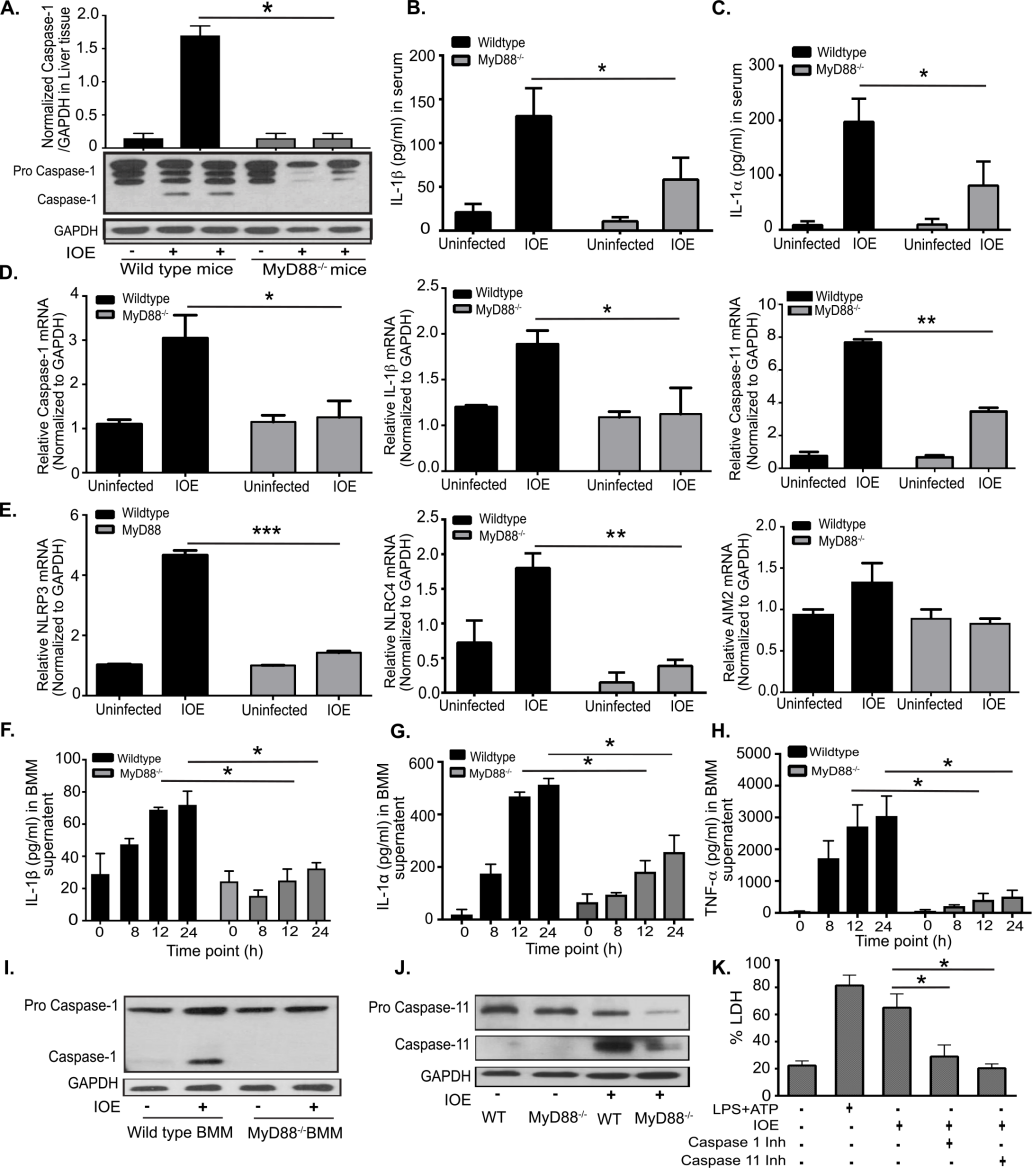
*Ehrlichia*-induced inflammasome activation is MyD88 dependent. (A) Western blot analysis of pro- and active/cleaved caspase-1 (p20) in whole liver lysates from uninfected and IOE-infected WT mice, compared with MyD88^-/-^ mice on day 7 p.i. GAPDH used as loading control. The density of bands in each group quantified and normalized to GAPDH expression. Levels of IL-1β (B) and IL-1α (C) in sera of naïve/uninfected and infected WT or MyD88^-/-^ mice on day 7 p.i. (D) mRNA expression of caspase-1, IL-1β and caspase-11 in liver tissues at day 7 p.i. in uninfected and IOE-infected WT and MyD88^-/-^ mice. (E) mRNA expression of NLRP3, NLRC4 and AIM2 in liver at day 7 p.i. WT and MyD88^-/-^ BMM were infected with IOE and the levels of IL-1β (F) and IL-1α (G) and TNF-α (H) in culture supernatants were measured at 0, 8, 12, and 24h post infection. (I) Expression of pro-caspase-1 and active/cleaved caspase-1 in uninfected and infected WT and MyD88^-/-^ BMM measured by immunoblotting at 24h p.i. (J) Expression of pro-caspase-11 and active/cleaved caspase-11 in uninfected and IOE-infected WT and MyD88^-/-^ BMM measured by immunoblotting at 24h p.i. (K) Level of LDH at 24h p.i. in uninfected or IOE-infected WT-BMM cultured in the presence/absence of caspase-1 inhibitor (Inh) or caspase-11 inhibitor (Inh). LPS+ATP were used as positive control. Data from *in vivo* experiments are from 3 mice/group and representative of three independent experiments. Data from *in vitro* experiments are representative of three independent experiments. All results presented as mean ± SD (* P<0.05, **P<0.01, ***P<0.001).

Since inflammasome activation is linked to severe liver injury in IOE-infected mice, we examined the contribution of MyD88 to inflammasome activation during IOE infection. To this end, we measured the expression of active (cleaved) caspase-1 in liver lysates from WT and MyD88^-/-^ mice by immunoblotting. Consistent with our previous studies [8, 24], IOE infection elicited inflammasome activation in the liver of WT mice as evidenced by cleaved caspase-1 in liver lysates (**Fig 2A**), and high serum levels of IL-1β on day 7 p.i. (**Fig 2B).** In contrast, MyD88 deficiency inhibited caspase-1 activation in the liver (**Fig 2A**), and decreased serum levels of IL-1β (**Fig 2B)** and IL-1α (a marker of lytic cell death induced by caspase-1/11-mediated pyroptosis) (**Fig 2C)** when compared to WT mice. At the transcription level, expression of caspase-1, IL-1β and caspase-11 were also lower in IOE-infected MyD88^-/-^ mice compared to WT mice (**Fig 2D**), similarly to inflammasome components NLRP3 and NLRC4 (**Fig 2E**). AIM2 levels did not change significantly with IOE infection, and were similar between WT and MyD88^-/-^ mice (**Fig 2E**).

Next we examined MyD88-mediated inflammasome activation in bone marrow derived macrophages (BMM) from WT and MyD88^-/-^ mice. Previous studies by us and other investigators suggest that most infected macrophages in the liver are derived from infiltrating blood monocytes during *Ehrlichia* infection[7, 44, 45], so using BMM rather than resident liver macrophages better recapitulates the *in vivo* cell types and response to *Ehrlichia*. WT and MyD88^-/-^ BMM were infected with IOE at multiplicity of infection MOI of 5 and the production of inflammasome-dependent and independent cytokines by infected macrophages was assessed at 8, 12, and 24h p.i. We selected these time points as they coincide with bacterial invasion and replication, without influencing cell viability. Consistent with the *in vivo* data, IOE induced significant secretion of inflammasome-dependent cytokines (IL-1β and IL-1α) and TLR-dependent, inflammasome-independent pro-inflammatory cytokine (TNF-α) in WT-BMM at 8, 12, and 24h p.i. In contrast, MyD88^-/-^ BMM have significantly lower production of IL-1β and IL-1α, and TNF-α at the same time points (**Fig 2F, 2G & 2H**). Decreased IL-1β secretion by IOE-infected MyD88^-/-^ BMM correlated with defective activation (cleavage) of caspase-1 (**Fig 2I**) and caspase-11 (**Fig 2J**) compared to WT-BMM, suggesting that IOE induces MyD88-dependent inflammasome activation. Further, the activation of inflammasome in WT macrophages was associated with lytic cell death as indicated by increased lactate dehydrogenase (LDH) (**Fig 2K**) release in infected BMM culture compared to uninfected BMM culture. Cell death of infected WT-BMM was significantly attenuated when cells were incubated with caspase-1 or caspase-11 inhibitors. Although these inhibitors may also inhibit other caspases, our data suggest that IOE-induces caspase-1/11-mediated pyroptosis (**Fig 2K**). As a positive control maximum LDH release was measured in response to stimulation of BMM with LPS+ATP (ligands for NLRP3 inflammasome).

To define the inflammasome complexes that contribute to IL-1β secretion following infection with virulent IOE and regulated by MyD88, we further analyzed mRNA expression of several inflammasome complexes (NLRP3, NLRC4, AIM2) in the liver tissues of WT and MyD88^-/-^ mice on day 7 p.i with IOE. IOE induced significant upregulation of NLRP3 in IOE-infected WT mice compared with infected MyD88^-/-^ mice. This result is consistent with our recent study showing that NLRP3 is an important inflammasome component during fatal *Ehrlichia* infection as it promotes IL-1β secretion and cell death of WT macrophages [24]. This is also consistent with a known role for MyD88 signaling in activation of NF-κB, which can then upregulate NLRP3 and pro-IL-1β message expression [46–48].

Although infected MyD88^-/-^ mice exhibited lower mRNA expression of NLRC4 than infected WT mice, NLRC4 mRNA expression in infected WT mice was only slightly elevated (less than 2 fold) compared to naïve WT or MyD88^-/-^ mice. These data are consistent with studies showing that *Ehrlichia* do not express the typical PAMPs known to activate NLRC4 such as flagella or type III secretion system effectors [22, 49], and suggests that NLRC4 is less likely to be important for inflammasome activation in our model.

Another main inflammasome that was of interest to our model is AIM2, which we previously found to be upregulated *in vivo* at early stages of lethal IOE infection (day 3 p.i.) [8]. In the current study, we did not detect significant differences in the level of AIM2 mRNA expression between IOE-infected WT and MyD88^-/-^ mice at late stages of infection (day 7 p.i.). However, our previous study has shown that AIM2 is upregulated in murine model of fatal ehrlichiosis at an early stage of infection (Day 3 p.i.). Thus, we examined whether AIM2 is critical for *in vivo* inflammasome activation and development of liver injury following lethal IOE infection. To this end, we infected WT and AIM2^-/-^ mice with an ordinarily lethal dose of IOE (10^4^ organisms/mouse). IOE-infected AIM2^-/-^ mice were susceptible to infection with 100% of the AIM2^-/-^ mice died on day 8–10 p.i. similar to WT mice (**S2A Fig**). H&E and TUNEL staining demonstrated liver damage in IOE-infected AIM2^-/-^ mice as marked by presence of several foci of liver necrosis and higher number of apoptotic macrophages and hepatocytes on day 7 p.i., when compared to infected WT mice (**S2B and S2C Fig**). Susceptibility of AIM2^-/-^ mice to lethal IOE infection correlated with high serum level of IL-1β and expression of active caspase-1 and IL-1β in liver tissues, which was similar to that detected in infected WT counterparts (**S2D and S2E Fig**). Together, these data suggest that AIM2 is neither critical for inflammasome activation during fatal *Ehrlichia* infection, nor responsible for development of *Ehrlichia*-induced liver injury or fatal toxic shock.

### MyD88 inhibits autophagy induction in *Ehrlichia*-infected macrophages

**Fig 3:**
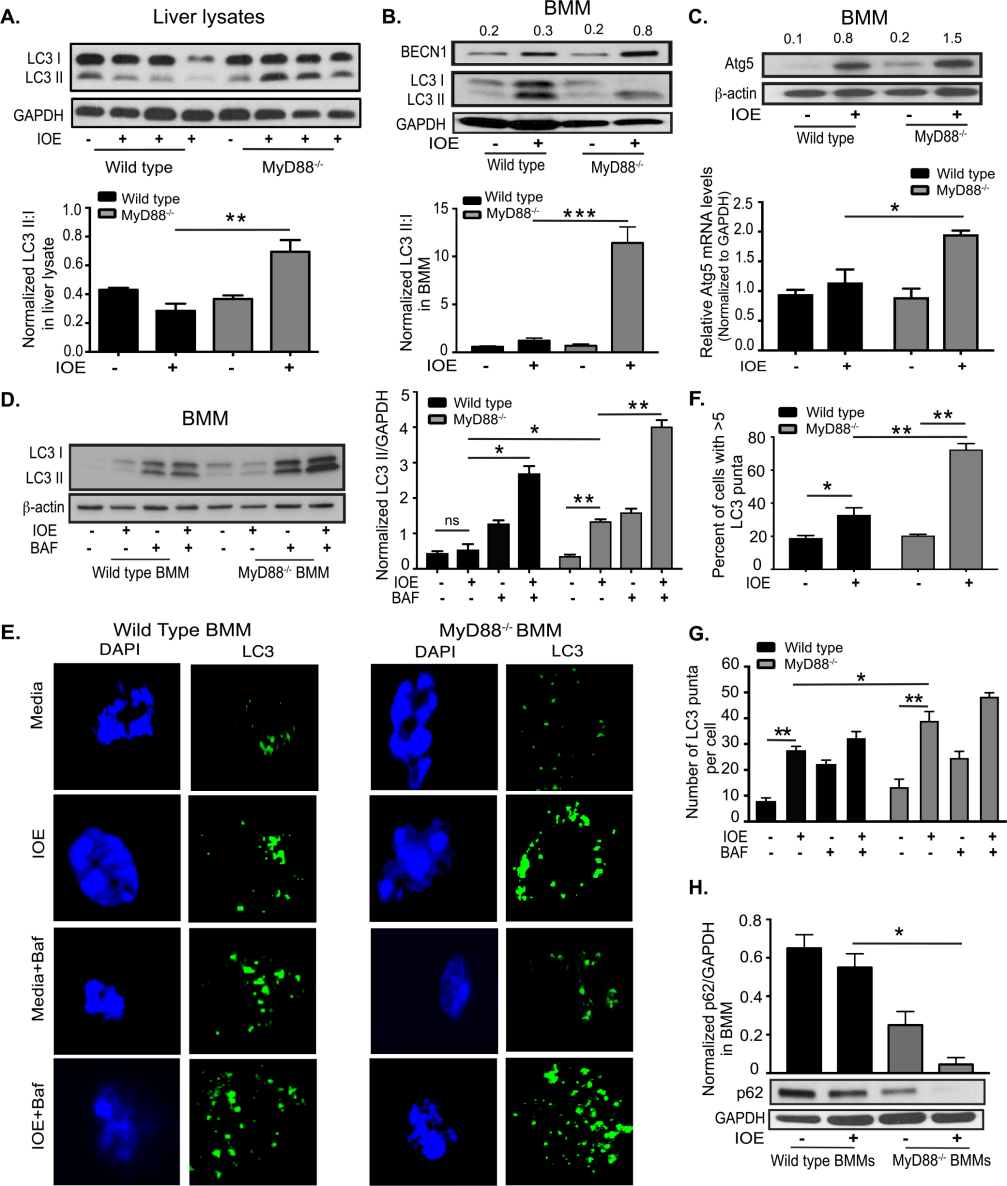
MyD88 inhibits autophagy *in vivo* and in *Ehrlichia*-infected macrophages. (A) Western blot of LC3I and LC3II in whole liver lysates from uninfected and IOE-infected WT and MyD88^-/-^ mice on day 7 p.i. Each lane represents one mouse and results representative of n = 9 per infected group and n = 9 of uninfected mice. The density of LC3II:LC3I bands in each group were quantified and normalized to GAPDH expression (lower graph). (B) Immunoblotting analysis of LC3I, LC3II and beclin-1 (BECN1) in uninfected and IOE-infected WT and MyD88^-/-^ BMM at 24h p.i. LC3II:I ratio was normalized to GAPDH (lower graph). (C) Immunoblotting analysis of Atg5 in uninfected and IOE-infected WT and MyD88^-/-^ BMM at 24h p.i. Normalized values for beclin 1 and Atg5 expression are indicated above the western blot bands. mRNA expression of Atg5 in uninfected and IOE-infected WT and MyD88^-/-^ BMM at 24h p.i. (D) Immunoblot of LC3I and LC3II in WT-BMM and MyD88^-/-^ BMM at 24h p.i. in the presence/absence of Baf. Quantification of LC3II band density normalized to GAPDH (middle graph). (E) Confocal immunofluorescent staining of LC3 (green) and nuclear DAPI staining (blue) in uninfected and IOE-infected WT and MyD88^-/-^ BMM treated with/without bafilomycin (Baf) at 24h p.i. (F) Quantification of percentage of cells with more than 5 LC3 puncta. (G) The number of LC3 puncta per cell quantified by confocal microscopy. (H) Immunoblot of p62 in uninfected and IOE-infected WT and MyD88^-/-^ BMM at 24h p.i. with p62 normalized to GAPDH loading control. All results are presented as mean ± SD (* P<0.05, **P<0.01, ***P<0.001) from three independent *in vitro* experiments. Quantification of immunoblots is presented as mean pixel density of bands/GAPDH from three mice/group and is representative of three independent experiments. All confocal images representative of at least three independent experiments and are representative of 10 high power fields (HPF).

Studies have shown that inflammasome activation is regulated by autophagy [48, 50]. To examine whether MyD88 mediates inflammasome activation via regulation of autophagy, we first determined whether IOE infection could trigger formation of autophagosomes, which entails the recruitment of cytosolic Atg8/LC3 to the phagophore [51]. Recruitment of non-lipidated LC3 (LC3I) to autophagosomes involves its proteolytic cleavage and lipidation, yielding LC3II. Therefore, to measure autophagy induction, we assessed conversion of LC3I to LC3II by immunoblot assay. Similar to uninfected WT mice, liver lysates from IOE-infected WT mice harvested on day 7 p.i. showed decreased accumulation of LC3II and a lower LC3II:I ratio **(Fig 3A).** In contrast, liver lysates from IOE-infected MyD88^-/-^ mice expressed more LC3II and a higher LC3II:I ratio when compared to naïve MyD88^-/-^ and infected WT mice (**Fig 3A**).

We next examined the autophagy process in macrophages as the major target cells for *Ehrlichia*. Autophagy levels were analyzed in primary BMM from WT or MyD88^-/-^ mice infected with IOE at MOI of 5. Cells were harvested at 24h p.i., and the autophagy was monitored by both immunoblotting and immunofluorescence using an anti-LC3 antibody. As shown in **Fig 3B**, IOE induced a significant increase of both LC3I and LC3II in WT-BMM compared to uninfected WT cells. However, the ratio of normalized LC3II: I, which reflects LC3II conversion/turnover associated with autophagosomes, was not significantly different than the LC3II: I ratio in naïve WT-BMM. In contrast, IOE-infected MyD88^-/-^BMM had higher accumulation of LC3II and thus higher LC3II: I ratio compared to uninfected MyD88^-/-^BMM and infected WT-BMM (**Fig 3B**). Further, expression of two major autophagy proteins that initiate autophagosome formation [27, 51]; beclin-1 (measured by immunoblot) and Atg5 (measured by immunoblot and RT-PCR) was higher in IOE-infected MyD88^-/-^ BMM when compared to WT-BMM at 24h p.i. (**Fig 3B & 3C**), suggesting that MyD88 impairs autophagy induction in IOE-infected macrophages.

We then examined whether the accumulation of LC3I and LC3II in WT and MyD88^-/-^ BMM was an early or late event during IOE infection. LC3 levels were analyzed in macrophages from both groups by confocal microscopy at different time points after IOE infection. Interestingly, we detected similar increases in LC3II puncta/cell (**S3A Fig**) as well as percentage of cells expressing more than 5 puncta (**S3B Fig**) in both WT- and MyD88^-/-^ BMM at early time points of post infection, being slightly induced at 4h and significantly increased by 8h, which corresponds with the entry of *Ehrlichia* into macrophages. However, at 12h and 24h post infection, we observed significant decrease in the number of LC3 puncta/cell, as well as the percentage of cells expressing more than 5 puncta, in IOE infected WT-BMM (**S3A and S3B Fig**). In contrast, LC3 puncta increased significantly in infected MyD88^-/-^ BMM at these later time points (**S3A and S3B Fig**). This difference correlates with the logarithmic replicating phase of *Ehrlichia* as previously described [52–54]. Since the differences in autophagy between WT- and MyD88^-/-^ BMM were mainly observed at 12h and 24h, we chose these time points for further analysis.

### MyD88 partially blocks autophagic flux in *Ehrlichia*-infected macrophages

The accumulation of LC3II in IOE-infected MyD88^-/-^ but not WT-BMM, could be due to enhanced autophagosome formation/induction, or a reduced degradation of autophagic cargo. To distinguish between these events, we analyzed autophagic flux in IOE-infected cells. To this end, we examined autophagosome-lysosomal fusion and lysosome-mediated autophagosome degradation by monitoring the effect of bafilomycin A1 (Baf: a lysosomal inhibitor of the vacuolar-type H+-ATPase) on the level of LC3II (immunoblot) and number of LC3 puncta (confocal), or the colocalization of autophagosomes with lysosomes (confocal). As shown by immunoblot, treatment of IOE-infected WT-BMM with Baf resulted in an increase of LC3II level [2.3 fold, calculated as the ratio between LC3 II level in IOE-infected WT-BMM treated with Baf (2.8) and those in untreated IOE-infected WT- BMM (0.5)] as compared with the effect promoted by the same treatment in uninfected cells, suggesting that LC3II degradation by lysosomal enzymes is enhanced during IOE infection of WT cells (**Fig 3D**). Similarly, treatment of IOE-infected MyD88^-/-^ BMM with Baf resulted in an increase of total LC3II [~3 fold, calculated as the ratio between LC3 levels in IOE-infected WT-BMM treated with Baf (4.4) and those in untreated IOE-infected WT- BMM (1.3)] as compared with the effect promoted by the same treatment in uninfected cells (**Fig 3D**), suggesting that LC3II degradation by lysosomal enzymes is further enhanced during IOE infection in the absence of MyD88. Confocal immunofluorescence analysis showed that IOE infection of WT-BMM and MyD88^-/-^ BMM significantly increased the percentage of cells with more than 5 LC3 puncta (**Fig 3F**), as well as the number of LC3 puncta/cell (**Fig 3E and 3G**) when compared to uninfected counterparts. IOE infection further increased the percentage of punctae and the number of LC3 puncta/cell in MyD88^-/-^ BMM treated with Baf (**Fig 3E, 3F and 3G**), which further confirm immunoblot data and suggest that MyD88 partially inhibits autophagic flux in IOE-infected BMM.

Partial block of autophagosome-lysosomal fusion or autophagosome degradation in WT-BMM, but not in MyD88^-/-^ cells, was further investigated by confocal microscopy examining the co-localization of LC3 puncta with acidic organelles (**S4A and S4B Fig**). To this end, cells were stained with an antibody against LC3 and also with LysoTracker Red, an acidotropic fluorescent dye that accumulates in acidic lysosomes. Compared to infected WT-BMM, MyD88^-/-^ BMM exhibited a significant increase in the number of LC3 puncta per cell that colocalized with the LysoTracker Red, suggesting formation of autolysosomes (**S4A and S4B Fig**). Decreased autolysosome formation in WT-BMM was not due to a block in lysosomal acidification by *Ehrlichiae* because the number of LysoTracker Red staining in IOE-infected WT and MyD88^-/-^ BMM was similar to LysoTracker Red staining in LPS-stimulated BMM from WT and MyD88^-/-^ mice, respectively (**S4A Fig**). Thus both confocal microscopy and immunoblotting analyses indicated that IOE induces MyD88-dependent partial block of the autophagosome-lysosomal fusion at late stage of the autophagic flux.

To further examine autophagic flux, we analyzed the level of p62/SQSTM1, a selective autophagy adaptor/receptor that binds to ubiquitinylated proteins and damaged organelles to target them to autophagosome-lysosomal compartments for degradation. The total cellular p62 expression levels inversely correlate with the autophagic flux and activity. Our data demonstrate a significant decrease in p62/SQSTM1 in IOE-infected MyD88^-/-^ BMM compared to uninfected MyD88^-/-^ BMM and infected WT-BMM (**Fig 3H**), confirming MyD88-mediated block of autophagic flux.

### MyD88-mediated inhibition of autophagy induction in infected macrophages is mediated via mTORC1 activation

**Fig 4:**
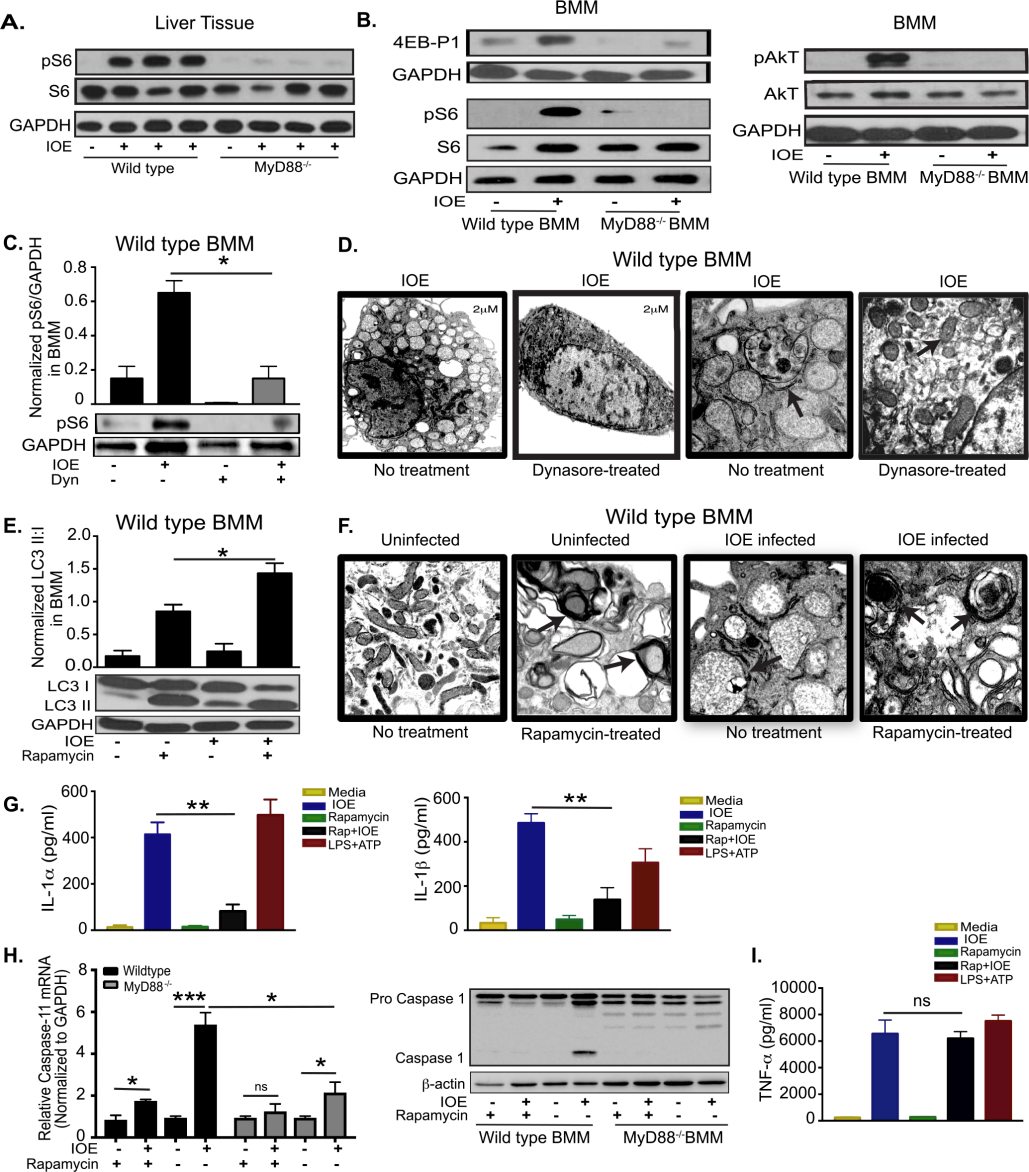
MyD88 inhibits autophagy induction and activates inflammasome via activation of mTORC1 pathway in macrophages. (A) Representative immunoblots of phosphorylation of S6 (pS6) and total S6 (S6) in whole liver lysates from uninfected and IOE-infected WT or MyD88^-/-^ mice on day 7 p.i. (B) Representative immunoblots showing expression of 4E-BP1, phospho- S6 (pS6), total S6 (S6), phospho-AKT (pAKT), and total AKT in uninfected and IOE-infected WT and MyD88^-/-^ BMM. (C) Representative Immunoblots showing phosphorylation of S6 (pS6) in WT-BMM infected with IOE and treated with/without Dynasore (Dyn). pS6 normalized to GAPDH at 24h p.i. (D) Transmission electron microscopy images of WT-BMM infected with IOE and treated with/without Dynasore at 24h p.i. Left two panels shows low magnification (scale bar 2μm), and right two panels show high magnification (scale bar 500nM) magnification, respectively. Arrow in no Dynasore-treated cells indicating *Ehrlichia* morula. Arrow on Dynasore-treated cell indicates normal mitochondrial morphology. (E) Immunoblotting analysis showing the expression of LC3I and LC3II in untreated and rapamycin-treated, uninfected and IOE infected WT-BMM at 24h p.i. The density of LC3II:LC3I bands were quantified and normalized to GAPDH loading control, and the ratio of normalized LC3II:I band is shown. (F) Transmission electron microscopy of uninfected (left two panels) and IOE-infected (right two panels) WT-BMM at 24h p.i., in the presence or absence of rapamycin showing higher formation of multiple double membrane autophagosomes in both uninfected and infected cells upon rapamycin treatment (Arrows). All panels are of high magnification (scale bar 500nM) (G) Concentrations of IL-1α and IL-1β in culture supernatant from uninfected and IOE-infected WT-BMM in the presence/absence of rapamycin at 24h p.i. LPS + ATP used as positive control. (H) mRNA expression of Caspase-11 and immunoblot of pro- and active/cleaved caspase-1 in uninfected and IOE-infected WT and MyD88^-/-^ BMMs in the presence/absence of rapamycin at 24h p.i. (I) TNF-α in culture supernatant from uninfected and IOE-infected WT-BMM infected with/without rapamycin treatment at 24h p.i. Negative controls included uninfected cells with or without rapamycin, while positive control included WT-BMM stimulated with LPS and ATP that activates NLRP3 inflammasome. All results are presented as mean ± SD (* P<0.05, **P<0.01, ***P<0.001) from three independent *in vitro* experiments. ns = not significant. Quantification of immunoblot bands is presented as mean pixel density of bands/GAPDH from three mice/group and is representative of three independent experiments.

To determine how MyD88 inhibits autophagy induction in macrophages, we examined the mTORC1 pathway as a major negative regulatory mechanism of autophagy. mTORC1 activation is measured by phosphorylation of its downstream targets ribosomal protein S6 (pS6) and eukaryotic initiation factor 4E-binding proteins (p4E-BP1), as well as phosphorylation of upstream AKT kinase. Compared to uninfected WT mice, IOE-infected WT mice had elevated pS6 in the liver tissue on day 7 p.i. (**Fig 4A**). In contrast, mTORC1 activation was significantly abrogated in the liver of IOE-infected MyD88^-/-^ mice **(Fig 4A).** Consistent with *in vivo* data, IOE also induced MyD88-dependent mTORC1 activation in macrophages as evidenced by increased expression of phosphorylated proteins p4E-BP1, pS6, and pAKT (levels of pS6 and pAKT were normalized to total S6 and AKT, respectively, as well as to GAPDH) in the *in vitro*-infected WT-BMM, but not in infected MyD88^-/-^ BMM, at 24h p.i. (**Fig 4B**). These data suggest that MyD88 promotes mTORC1 activation in macrophages following IOE infection.

To determine whether intracellular *Ehrlichiae* or other host factors are responsible for activating MyD88-mediated mTORC1 activation, we blocked bacterial internalization in WT-BMM with the dynamin inhibitor, dynasore. Dynasore treatment attenuated expression of pS6 (**Fig 4C**) and cellular damage as indicated by restoration of normal mitochondrial structure in dynasore-treated infected cells compared to untreated IOE-infected WT-BMM (**Fig 4D).** Thus, these data indicate that MyD88-mediated activation of mTORC1 in IOE-infected macrophages is likely due, in part, to bacterial PAMPs.

To further confirm the role of mTORC1 activation in IOE-induced autophagy inhibition, we treated IOE infected WT-BMM with rapamycin (mTORC1 inhibitor that stimulates autophagy), and examined LC3II accumulation. As shown in **Fig 4E,** rapamycin treatment of IOE-infected WT-BMM resulted in significant LC3II turnover, resulting in a higher LC3II:I ratio compared to untreated infected cells. These results were also confirmed by electron microscopy, which demonstrated increased autophagosome formation in both uninfected and IOE-infected BMM upon rapamycin treatment **(Fig 4F).** Interestingly, rapamycin treatment of IOE-infected WT-BMM attenuated production of both IL-1α and IL-1β (**Fig 4G**). This correlated with decreased mRNA expression of caspase-11 as measured by RT-PCR, as well as decreased caspase-1 activation measured by immunoblotting in IOE-infected WT-BMM when compared to untreated/infected WT-BMM (**Fig 4H**). Notably, infected MyD88^-/-^ BMM, cultured with or without rapamycin had decreased expression of caspase-11 mRNA and did not express active caspase-1 when compared to infected WT-BMM with and without rapamycin, respectively (**Fig 4H**). The effect of rapamycin on the production of inflammasome-dependent cytokines was not due to generalized immunosuppressive effects of rapamycin since it did not influence the secretion of TNF-α (a TLR/NF-κB-dependent cytokine) (**Fig 4I**). These data suggest that mTORC1 activation may enhance inflammasome activation in macrophages following IOE infection via inhibition of autophagy induction.

### MyD88-mediated inhibition of autophagy induction is host-protective mechanism.

**Fig 5:**
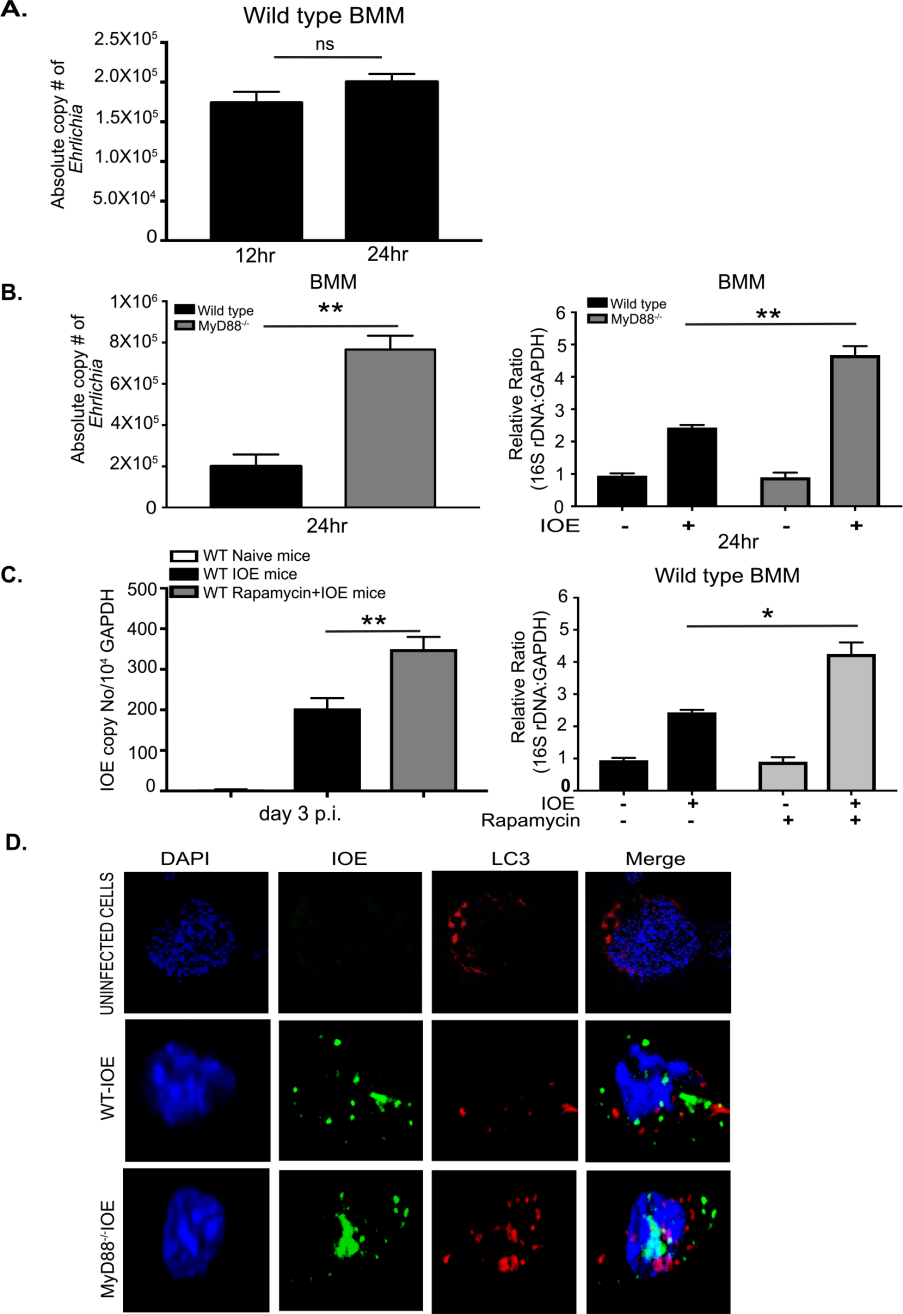
IOE exploits autophagy for its survival and replication. (A) Absolute number of intracellular bacteria in WT-BMM at 12 and 24h p.i. (B) Absolute number of intracellular bacteria in BMM from WT and MyD88^-/-^ mice infected with IOE at 24h p.i. Right graph shows relative expression of IOE 16S rDNA, detecting viable bacteria, normalized to GAPDH in uninfected and infected WT and MD88^-/-^ BMM at 24h p.i. (C) Left panel show bacterial burden in the livers of naïve/uninfected and IOE-infected WT mice, with/without rapamycin treatment, on day 3 p.i. Right panel shows that the number of viable IOE analyzed by 16S rDNA RT-PCR in uninfected and infected WT-BMM with/without rapamycin treatment at 24h p.i. (D) Immunofluorescence staining of IOE organisms (green staining) LC3 (red staining) and DAPI nuclear stain (blue staining) in uninfected and IOE-infected WT and MyD88^-/-^ BMMs at 24h p.i. Data showing lack of colocalization between LC3 and bacteria in both WT and MyD88^-/-^ macrophages. *In vivo* data are presented as mean ±SD of 3 mice/group and representative of 3 independent experiments. *In vitro* data representative of 3 independent experiments and results are presented as mean ± SD (* P<0.05, **P<0.01). ns = not significant.

To determine the effect of autophagy regulation by MyD88 on host defense against IOE, we measured intracellular *Ehrlichia* in WT and MyD88^-/-^ BMM by qPCR. At 12h and 24h p.i., cells were washed twice to remove extracellular *Ehrlichia*, and the number of intracellular bacteria was determined by qPCR. Our data show that the number of ehrlichiae within WT-BMM at 24h p.i. was comparable to the number of intracellular *Ehrlichia* at 12h p.i. (**Fig 5A**). Since the intracellular bacterial number is the net result of bacterial killing and replication, these data suggest bacteria may not be effectively replicating within WT macrophages or that they are eliminated by intracellular bactericidal mechanisms of macrophages. On the other hand, the number of viable *Ehrlichia* as determined by qPCR and RT-PCR of *dsb* and 16S rRNA, was significantly higher in the MyD88^-/-^ BMM when compared to WT-BMM at 24h p.i. (**Fig 5B**). These data suggest that MyD88 signaling in macrophages is critical for inhibition of bacterial survival and/or replication. To examine the effect of autophagy on bacterial survival and replication *in vivo*, we treated IOE-infected WT mice with rapamycin during the early stage of infection (day 1–3 p.i.) and measured bacterial burden on day 3 p.i. Interestingly, rapamycin treatment significantly increased IOE bacterial burden in the liver compared to untreated infected mice (**Fig 5C**). Consistent with the *in vivo* data, rapamycin treatment (inducer of autophagy) of IOE-infected WT-BMM resulted in a significant increase in the number of viable intracellular *Ehrlichia* as measured by 16S rRNA at 24h p.i. when compared to infected untreated WT-BMM or uninfected WT-BMM treated with or without rapamycin (**Fig 5C**). Together, these results suggest that MyD88-mediated inhibition of autophagy induction is also a host-protective mechanism limiting survival and/or replication of IOE.

Since lack of MyD88 enhances colocalization of LC3 and lysosomes, we asked the question why replicating intracellular *Ehrlichia* in MyD88^-/-^ BMM are not effectively eliminated. To address this question, we analyzed the colocalization of *Ehrlichia* with LC3 and lysosomes in both WT and MyD88^-/-^ BMM. Our data demonstrate that IOE did not colocalize with LC3 (**Fig 5D**) or lysosomes (**S5 Fig**) in WT or MyD88^-/-^ macrophages. These data are consistent with recent studies showing defective co-localization of LC3 with a closely related *Ehrlichia* species, *E*. *chaffeensis* [37, 55], and this does not appear to be MyD88-dependent.

### Activation of mTORC1 and inflammasome is mediated by TLR9

**Fig 6:**
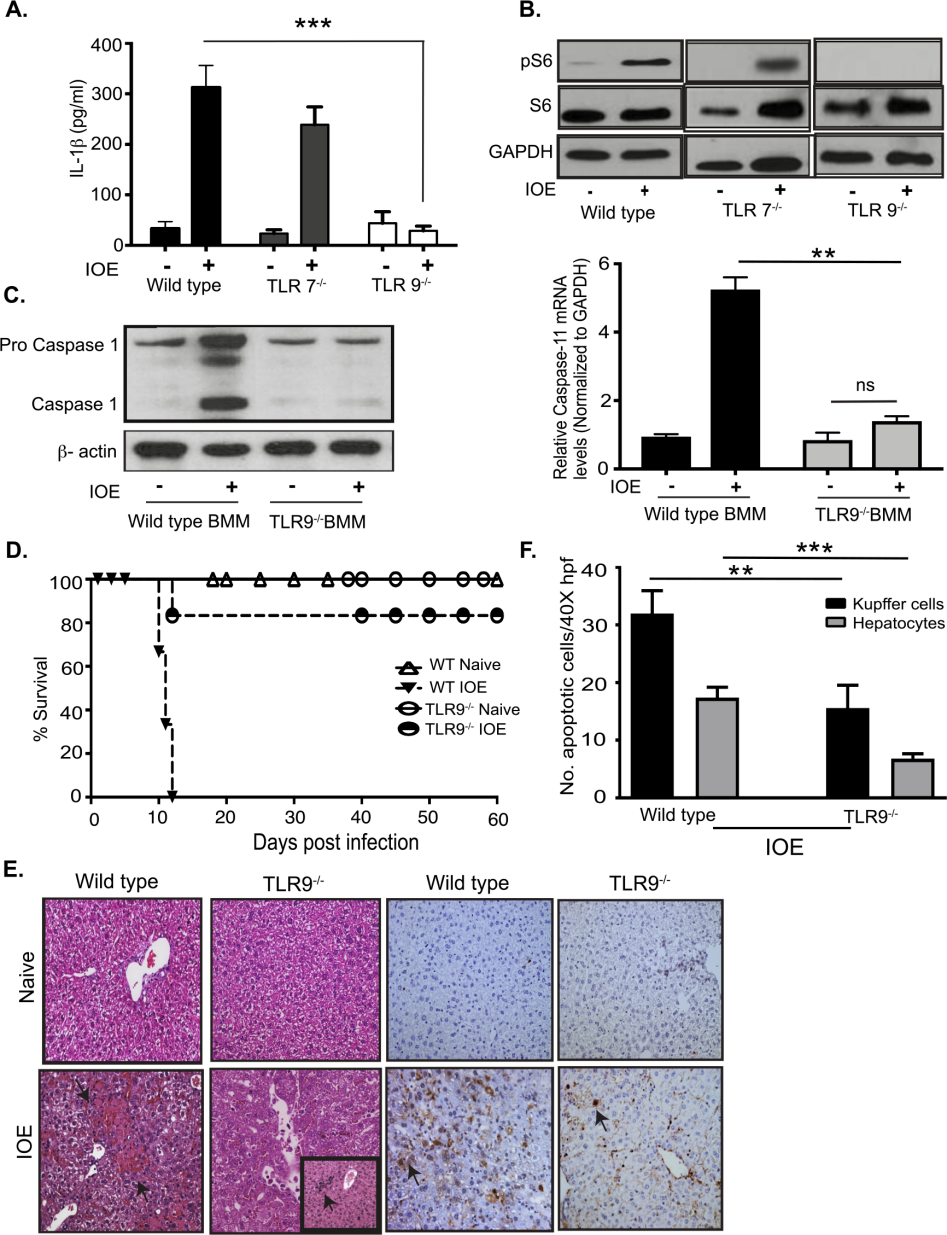
TLR9-dependent inflammasome and mTORC1 activation in IOE infected macrophages. A) IL-1β in cell culture supernatants from uninfected and IOE infected (MOI 5) WT, TLR7^-/-^, and TLR9^-/-^ BMM at 24h p.i. (B) Representative Immunoblot analyzing phosphorylation of phospho S6 (pS6) in WT, TLR7^-/-^, and TLR9^-/-^ BMM at 24h p.i. GAPDH used as loading control. (C) Immunoblot analysis of Caspase-1 in WT and TLR9^-/-^ BMM at 24h p.i. β-actin used as loading control. mRNA expression of Caspase-11 in infected WT and TLR9^-/-^ BMM and controls at 24h p.i. (D) Survival of uninfected and IOE-infected WT and TLR9^-/-^ mice. Data showing 85% survival of IOE infected TLR9^-/-^ mice compared to WT mice till day 60 p.i. (n = 6/group). (E) Representative H&E and TUNEL staining of liver sections from naïve/uninfected and IOE-infected WT and TLR9^-/-^ mice on day 7 p.i. The insert in the H&E staining of liver section from IOE-infected TLR9^-/-^ mice demonstrates an enhanced cellular infiltration consistent with regenerative changes. (F) Quantification of TUNEL positive Kupffer cells and hepatocytes per high power field (HPF) in TLR9^-/-^ and WT mice. Data from *in vitro* experiments are representative of three independent experiments. All results are presented as mean ± SD (* P<0.05, ***P<0.001).

Virulent IOE infection triggers upregulation of several TLRs in liver tissue of infected mice including TLR2, TLR7, and TLR9 [8]. To determine which TLR signal activates MyD88- mediated mTORC1 and inflammasome activation, we measured mTORC1 activation and production of IL-1β in BMM from TLR7^-/-^ and TLR9^-/-^ mice. We focused on endosomal TLRs as *Ehrlichia* are located intracellularly within endosomes. IOE infection of TLR7^-/-^ BMM elicited IL-1β at slightly lower levels when compared to WT-BMM (**Fig 6A**). On the other hand, lack of TLR9 signaling significantly attenuated the secretion of IL-1β following IOE infection in TLR9^-/-^ BMM (**Fig 6A**), and abrogated S6 phosphorylation (**Fig 6B**). Reduced secretion of IL-1β in IOE-infected TLR9^-/-^ BMM correlated with reduced expression of active caspase-1 and caspase-11 mRNA compared to infected WT-BMM **(Fig 6C)**. To directly investigate the role of TLR9 in *Ehrlichia*-induced liver injury and toxic shock, we infected WT and TLR9^-/-^ mice with an ordinarily lethal dose of IOE. Interestingly, TLR9^-/-^ mice were highly resistant to lethal ehrlichiosis as evidenced by 85% survival of mice (n = 6) until day 60 p.i., while 100% of WT mice succumbed to infection on days 10–12 p.i. (**Fig 6D).** Protection of TLR9^-/-^ mice against fatal toxic shock was associated with significant attenuation of liver injury as evidenced by decreased cell necrosis and fatty changes/steatosis, as well as enhanced cellular infiltration as measured by H&E staining (**Fig 6E).** TUNEL staining also revealed decreased number of TUNEL positive apoptotic hepatocytes and Kupffer cells in IOE-infected TLR9^-/-^ mice (**Fig 6E and 6F**) at day 7 p.i., when compared to WT mice. Together, these data suggest TLR9 is a key upstream pattern recognition receptor (PRR) mediating MyD88-dependent activation of mTORC1 and inflammasome during *Ehrlichia* infection.

### MyD88 is required for type I IFN-mediated inflammasome activation

**Fig 7:**
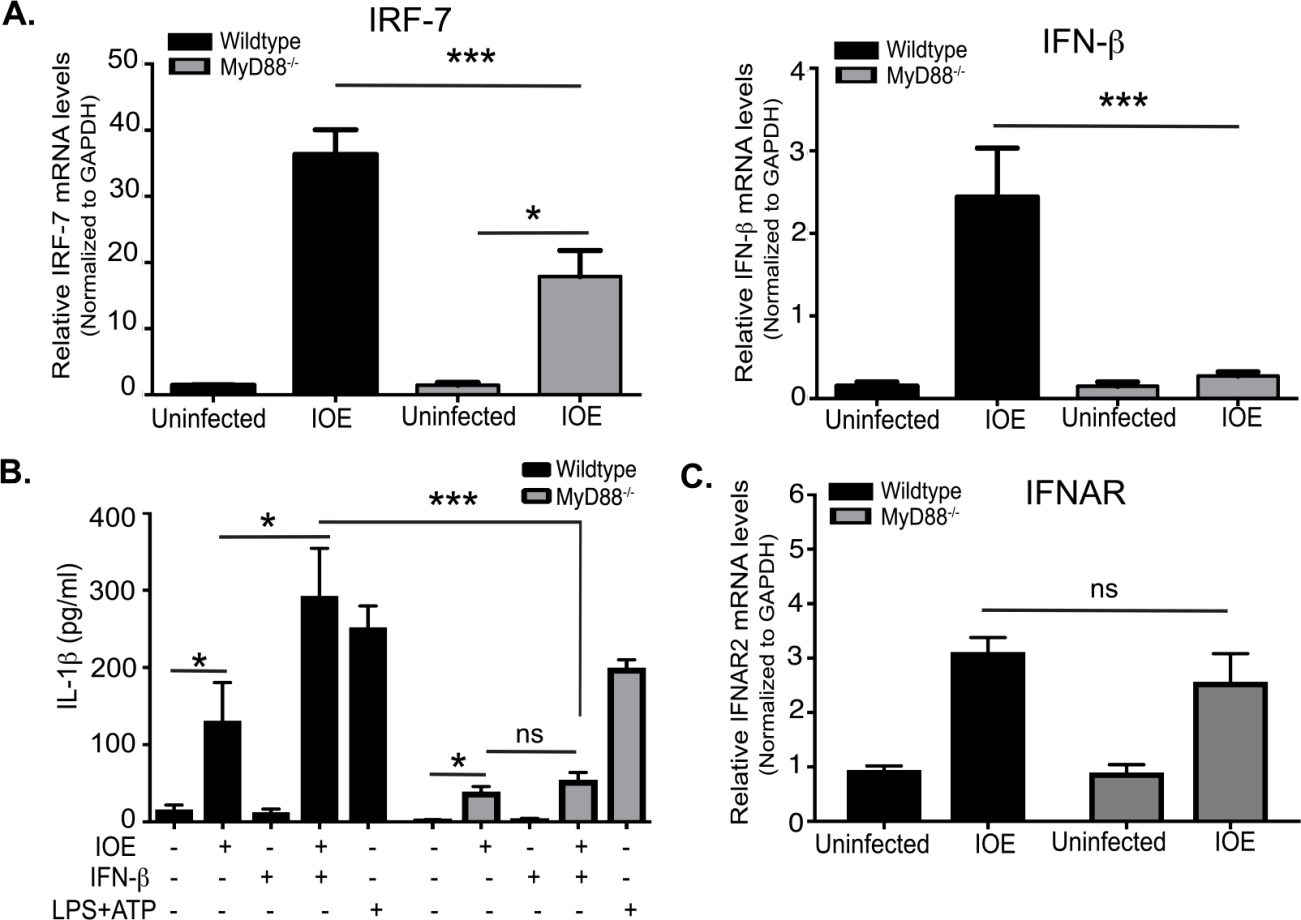
MyD88 is required for type I IFN-mediated inflammasome activation. (A) mRNA expression of IRF-7 and IFNβ in uninfected and IOE-infected WT and MyD88^-/-^ BMM at 24h p.i. (B) Level of IL-1β in cell culture supernatants of uninfected and IOE-infected WT and MyD88^-/-^ BMM in the presence/absence of IFNβ (500 IU/ml) at 24hr p.i. LPS + ATP is used as positive control. (C) The relative mRNA expression of IFNAR in uninfected and IOE-infected WT and MyD88^-/-^ BMM normalized to GAPDH at 24h p.i. All results are presented as mean ± SD (* P<0.05, ***P<0.001) from three independent *in vitro* experiments. ns = not significant.

We and others have shown that fatal IOE infection induces secretion of IFN-I cytokines, mainly by monocytes and plasmacytoid dendritic cells [8, 24, 32]. Binding of IFN-I cytokines to IFNAR induces multiple downstream signaling pathways that lead to diverse biological effects [56–59]. Our recent study showed IFNAR signaling induced caspase-11 activation, host cell death and detrimental inflammasome activation [24]. Since the host responses in IOE-infected MyD88^-/-^ mice partially phenocopies the response in IOE-infected IFNAR^-/-^ mice described in our previous study [24], we hypothesized that MyD88 signaling may regulate IFN-I response in macrophages. To this end, we analyzed mRNA expression levels of interferon regulatory factor 7 (IRF7), which regulates transcription of IFN-I genes, as well as IFNβ in IOE-infected WT and MyD88^-/-^ BMM. Consistent with previous *in vivo* data [8], IOE infection enhanced expression of IRF7 and IFNβ in WT-BMM **(Fig 7A),** but not in MyD88^-/-^ BMM. This suggests that IFNβ production in macrophages is downstream of MyD88 signaling during fatal ehrlichial infection.

To further determine whether inflammasome activation can be rescued in infected MyD88^-/-^ BMM by addition of IFNβ, we treated BMM from WT and MyD88^-/-^ mice with or without IFNβ followed by IOE infection, and measured levels of IL-1β. Stimulation of WT-BMM with LPS and ATP (positive control for NLRP3 inflammasome) induced a significantly higher production of IL-1β compared to unstimulated WT cells. Lack of MyD88 signaling partially decreased IL-1β production by both LPS treated and IOE-infected cells, suggesting that LPS/ATP and IOE induce inflammasome activation via MyD88-dependent and independent pathways (**Fig 7B**). Notably, the addition of IFNβ to IOE-infected WT-BMM, but not MyD88^-/-^ BMM, induced higher levels of IL-1β secretion when compared to uninfected and untreated/infected BMM controls (**Fig 7B).** Failure of IFNβ to rescue IL-1β production in MyD88^-/-^ BMM was not associated with significant difference in mRNA expression of IFNAR in these cells when compared to WT-BMM (**Fig 7C**). Since MyD88 is critical for transcription and upregulation of pro-IL-1β and pro-caspases-1/11 as shown in Fig 2, these data suggest that IFN I- mediated inflammasome activation during IOE infection requires priming by MyD88.

### Virulent *Ehrlichia* induces mitochondrial damage and p62/SQSTM1 accumulation in macrophages.

**Fig 8:**
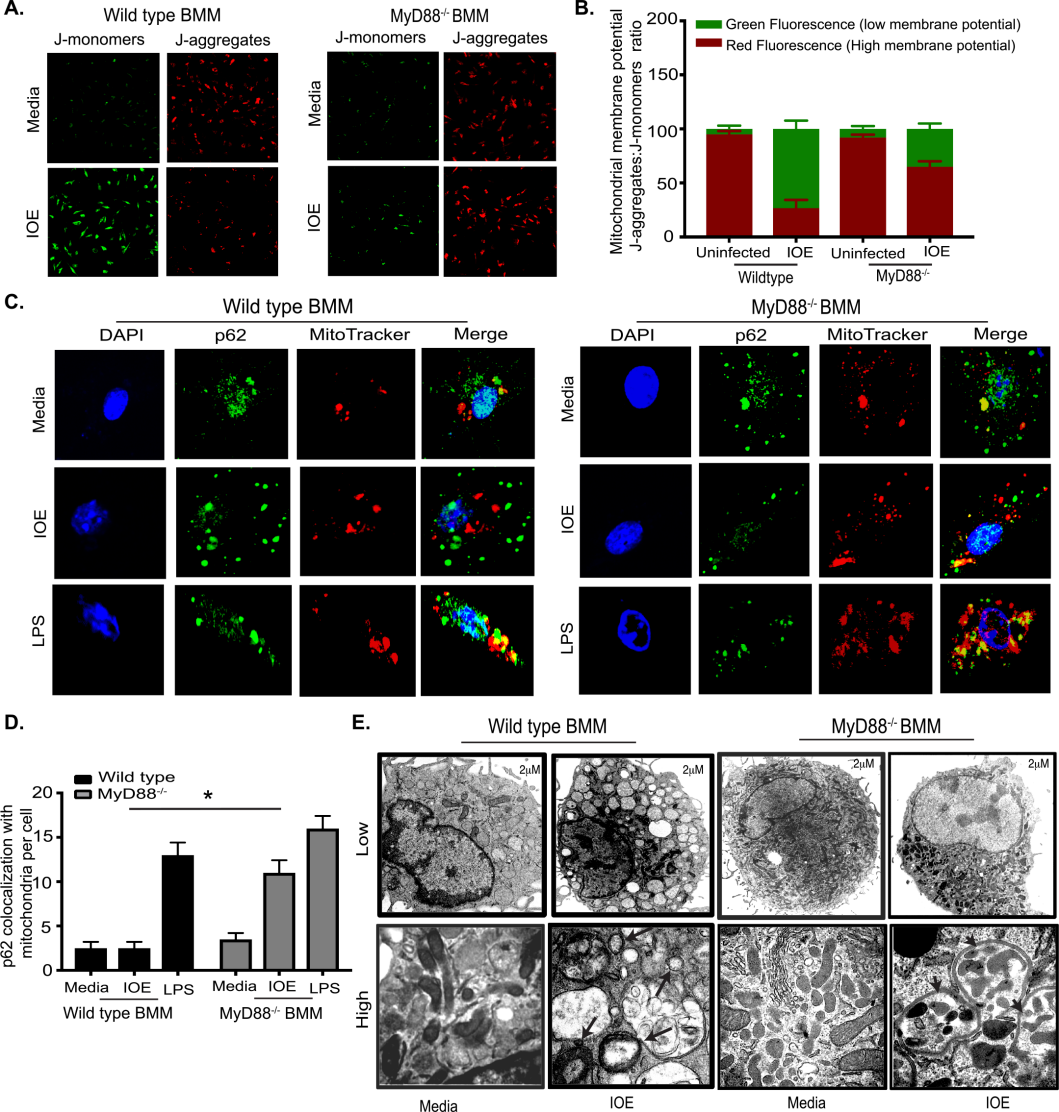
Virulent *Ehrlichia* induce mitochondrial damage and inhibit mitochondrial clearance. (A) Confocal images of JC-1 staining in naïve and IOE infected WT and MyD88^-/-^ BMM at 24h p.i. Red fluorescence indicates healthy mitochondrial J-aggregates and green indicates J-monomers and level of mitochondrial dysfunction. (B) Quantification of JC-1 ratio of aggregates: monomers. (C) Confocal immunofluorescence colocalization (yellow) of p62 (green) with MitoTracker (red) and DAPI nuclear stain in uninfected (media) and IOE-infected WT (left) and MyD88^-/-^ (right) BMM at 24h p.i. Uninfected (media) BMM and LPS (200 ng/ml) was used as negative and positive controls, respectively. (D) Quantification of p62 colocalization with MitoTracker per cell. Data are presented as mean ± SEM from three different experiments. *P<0.05. (E) Transmission electron microscopy images of uninfected and IOE-infected WT and MyD88^-/-^ BMM at 24h p.i. Upper panels shows low magnification (scale bar 2μm); lower panels show high magnification (scale bar 500nM). Vacuoles resembling enlarged lysosome and damaged swollen mitochondria (arrows). Autophagosomes surrounding mitochondria (arrow heads). Data are from three independent experiments. All results are presented as mean ±SD (* P<0.05).

TLR9 signaling is known to be triggered by either bacterial DNA or mitochondrial (mt) DNA. Based on the above data showing that TLR9 mediates IOE-induced activation of mTORC1 and inflammasome in macrophages and is a key mediator of *Ehrlichia*-induced liver injury, we hypothesized that TLR9/MyD88 pathway and subsequent inflammasome activation in infected macrophages could be triggered by mt DNA that accumulates in infected cells as a result of inhibition of mitochondrial autophagy (mitophagy). To examine this hypothesis, we first analyzed the mitochondrial membrane permeability, as an important parameter of mitochondrial function, in infected WT and MyD88^-/-^ BMM using JC-1 dye and confocal microscopy. We stained WT and MyD88^-/-^ BMM infected with IOE for 24h with JC-1 for 30min. Uninfected BMM from both mice groups were stained at the same time points as controls. JC-1 monomers aggregate on the mitochondrial surface, which shows as red fluorescence in healthy cells with high mitochondrial potential. On the other hand, JC-1 remains in the monomeric form in apoptotic or unhealthy cells with low mitochondrial potential, which shows as green fluorescence. Our data demonstrate a significantly higher ratio of fluorescent intensity of JC-1 monomers (green fluorescence) to fluorescent intensity of aggregates (red fluorescence) in IOE-infected WT-BMM, indicating a predominance of cells with low mitochondrial potential consistent with damaged mitochondria (**Fig 8A and 8B**). In contrast, red-fluorescing, highly energized mitochondria were proportionally more prevalent in IOE infected MyD88^-/-^ BMM, suggesting healthier cells with high mitochondrial potential (**Fig 8A and 8B**).

To further determine whether damaged mitochondria are eliminated, we analyzed colocalization of p62 with damaged mitochondria as evidence of enhanced mitophagy. To this end, cells were stained with an antibody against p62 and also with MitoTracker, a fluorescent dye that stains healthy and damaged mitochondria. Similar to immunoblotting data in (**Fig 3H**), immunofluorescence staining of IOE-infected WT-BMM demonstrated higher p62 expression than MyD88^-/-^ BMM. However, p62 failed to colocalize with MitoTracker in IOE-infected WT-BMM compared to p62-MitoTracker colocalization in LPS-treated WT-BMM (**Fig 8C**). On the other hand, there was significantly higher double positive p62-Mitotracker staining in IOE-infected MyD88^-/-^ BMM when compared to WT-BMM, suggesting a better colocalization of p62 with damaged mitochondria and enhanced mitophagy in MyD88^-/-^ BMM (**Fig 8C and 8D**). Additionally, electron microscopy analysis indicated that IOE-infected WT-BMM have few double-membrane autophagosomes and autolysosome, but many vacuoles resembling swollen lysosomes or damaged mitochondria (**Fig 8E**). In contrast, IOE-infected MyD88^-/-^ BMM had healthier mitochondrial morphology, and cells contained several autolysosomes containing mitochondria (**Fig 8E**).

**Fig 9:**
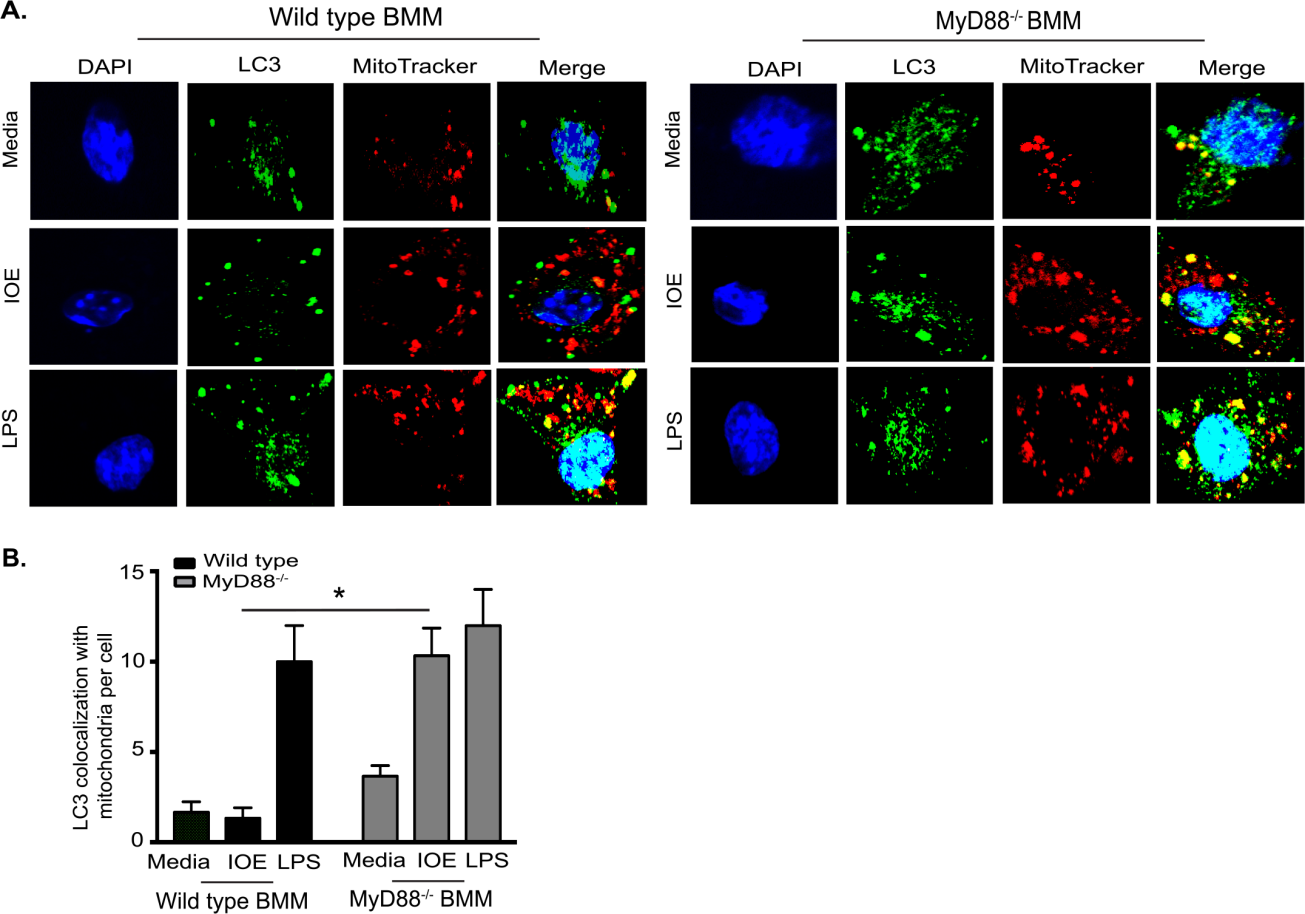
MyD88 inhibits mitophagy in macrophages. **(A)** Representative confocal immunofluorescence colocalization (yellow) of LC3 (green) and mitochondria (MitoTracker; red), and DAPI nuclear stain (blue) in uninfected (media) and IOE-infected WT and MyD88^-/-^ BMM at 24h p.i. Cells stimulated with LPS (200ng/mL) were used as positive controls. (B) Quantification of LC3 colocalization with MitoTracker per cell. Data are presented as mean ± SEM from three different experiments. *P<0.05.

To further examine whether damaged mitochondria that colocalize with p62 are effectively eliminated via the autophagy flux in MyD88^-/-^ BMM, we analyzed colocalization of LC3/autophagosomes with mitochondria. Our data show that IOE-infected MyD88^-/-^BMM has increased LC3 colocalization with mitochondria, while IOE-infected WT-BMM has little or no colocalization of LC3 with mitochondria **(Fig 9A and 9B)**. These data suggest that MyD88-mediated inhibition of autophagy results in defective mitophagy and accumulation of mitochondrial DAMPs. Although the contribution of *Ehrlichia*-derived PAMPs to inflammasome activation needs to be investigated, these data suggest that mitochondrial DAMPs are potential ligands for TLR9/MyD88-mediated inflammasome activation during lethal infection with LPS-negative *Ehrlichia*.

## Discussion

Liver is the major target organ in human monocytic ehrlichiosis. HME patients develop severe liver injury marked by an initial elevation of liver transaminases followed by progressive hepato-splenomegaly, hepatic lobular lympho-histiocytic cholestasis, foamy and activated macrophages, and bile duct epithelial injury, which progress to liver failure [60]. Our study demonstrates, for the first time, a novel mechanism whereby virulent *Ehrlichia* (IOE) causes liver damage via the MyD88-mTORC1 pathway. Our data indicate that *Ehrlichia* activates the MyD88-mTORC1 pathway in macrophages to induce host-pathogenic inflammasome activation. Mechanistically, these events occur via MyD88-mediated inhibition of autophagic induction and flux as well as blocking mitophagy leading to accumulation of inflammasome activators including PAMPs and DAMPs. We have previously shown that severe liver injury in the fatal murine model of ehrlichiosis is caused by IL-18 and IL-1β-mediated expansion of pathogenic cytotoxic CD8^+^ T cells and neutrophils [26]. These cells not only induce death of infected cells, but also induce death of CD4^+^ Th1 cells that are important for protective immunity against *Ehrlichia* via mechanisms that involve TNF/TNFR and FAS signals. In this study, lack of MyD88 decreased the frequency of activated CD8^+^ T cells and increased the number of CD4^+^ T cells, which could also account for the attenuated liver immunopathology at late stages of infection.

Autophagy is a critical innate immune host defense mechanism against several facultative intracellular bacterial pathogens such as *Legionella* and *Salmonella* [61–64]. However, the role of autophagy in protective innate immunity against infection with obligate intracellular virulent bacteria such as *Ehrlichia* is not clearly defined. Our study suggests that autophagy promotes survival and/or replication of *Ehrlichia*, and that MyD88 signaling impairs bacterial replication by inhibiting autophagy induction. This conclusion is supported by the finding that enhanced autophagy induction *in vivo* in IOE-infected MyD88^-/-^ mice and rapamycin-treated/infected WT mice as well as its corresponding *in vitro* validation in macrophages promoted bacterial survival and replication (**Fig 1 and Fig 5**). A recent study by Lin et al.[37], indicated that autophagy provides amino acids to *Ehrlichia* that enabled their survival and replication and that blocking autophagy induction using 3-MA treatment or knockdown of Atg5 or beclin-1 attenuates bacterial growth [37, 38]. Thus, the high bacterial burden in MyD88^-/-^ mice and macrophages, as major target cell for *Ehrlichia*, is likely due to ability of IOE to hijack nutrients via autophagy proteins. Although MyD88 deficiency enhanced autophagosome-lysosome fusion in macrophages, the lack of colocalization of IOE organisms with the LC3II/autophagsomes and lysosomes suggest that the replicating bacteria in MyD88^-/-^ cells cannot effectively be eliminated.

Several studies in mice and humans reported the negative regulation of inflammasome by autophagy. Genetically or pharmacologically defective autophagy in monocytes leads to accumulation of damaged mitochondria and increased concentration of mitochondrial DAMPs such as reactive oxygen species (ROS) and oxidized mitochondrial DNA (mtDNA). Robust generation of mt ROS or mtDNA and their release into cytosol causes activation of cytosolic NLRP3 inflammasome complex, which in turn cleaves pro-caspase-1 or pro-caspase-11 and leads to cleavage of mature IL-1β and IL-18. Our study highlights a novel positive role of MyD88 in regulation of inflammasome activation via two pathways. *First*, MyD88 signaling induces NF-κB activation and subsequent expression of pro-IL-1β and other inflammasome components, which provides “signal 1” for inflammasome activation. This conclusion is supported by our data showing reduced production of NF-κB-dependent TNF in MyD88^-/-^ cells as well as decreased mRNA and protein levels of NLRP3/NLRC4 and pro-caspase-1/11 (**Fig 2**). *Second*, MyD88-mediated inhibition of autophagy induction and flux leads to defective mitophagy and accumulation of damaged mitochondria, which in turn results in release of mtDNA or other mitochondrial DAMPs (e.g., ROS or oxidized cardiolipin). These mt DAMPS act as “signal 2” triggering inflammasome activation. This conclusion is supported by TEM analysis showing MyD88-dependent accumulation of swollen damaged mitochondria (**Fig 8E**), which we further confirmed by confocal microscopy analysis showing lack of colocalization between p62 and damaged mitochondria (**Fig 8C and 8D**) as well as lack of colocalization between LC3 and mitochondria (**Fig 9A and 9B**) in WT-BMM, but not in MyD88^-/-^ BMM. This clearly demonstrates that IOE induces mitochondrial damage and block mitophagy in macrophages via MyD88 signaling. These data are consistent with recent studies showing that infection of macrophages with *E*. *chaffeensis*, a human pathogen closely related to IOE, inhibits mitochondrial metabolism [54, 65]. The mechanism that account for enhanced mitophagy in MyD88^-/-^, but not in WT, macrophages remains elusive. One mechanism by which mitophagy is induced is through Parkin/PINK1 where PINK1 binds selectively to damaged mitochondria and oxidized cardiolipin, which leads to the recruitment of Parkin (E3 ubiquitin ligase) to mitochondria and ubiquitination of mitochondrial substrates. This process is followed by recruitment of the ubiquitin-binding adaptor p62, which deliver the parkin-ubiquitylated cargo into autophagosomes for degradation by binding to LC3. Future studies will examine whether a defect in the PINK1-Parkin-Cardiolipin-p62 pathway is responsible for defective mitophagy in WT macrophages following infection with virulent *Ehrlichia*.

This study strongly indicates that MyD88 is a key regulator of inflammasome activation. However, the data showing partial decrease in serum levels of IL-1β and IL-1α in IOE-infected MyD88^-/-^ mice (Fig 2), suggest a potential role of MyD88-independent pathways such as TIR-domain-containing adaptor-inducing interferon-β (TRIF) and type-I IFN receptor signaling pathways in IOE-induced inflammasome activation. In support of MyD88-independent pathway (s), we found that infected TLR9^-/-^ mice are completely protected against lethal ehrlichiosis compared to partial protection of infected MyD88^-/-^ mice (**Fig 6**). Lack of TLR9 signaling in macrophages also completely abrogated canonical and non-canonical inflammasome activation. These data suggest that TLR9 is the major PRR causing inflammasome activation, subsequent liver damage, and fatal toxic shock following lethal *Ehrlichia* infection. TLR9 signaling is known to trigger two downstream pathways. The first pathway leads to transcriptional activation of NF-κB-dependent proinflammatory cytokines, and the second pathway leads to the activation of IFN-I genes through phosphorylation of IRF7[66, 67]. Although both pathways are MyD88-dependent, the IFN-I pathway and responses also requires additional signaling[59]. We and others have previously showed that IFNAR signaling is pivotal not only for inflammasome activation and secretion of IL-1β, but also for development of IOE-induced toxic shock, via mechanisms that involve caspase-11 activation [23, 24, 32]. In the current study, we show that IFNβ promotes IL-1β secretion by IOE-infected WT macrophages compared to untreated/ infected cells (**Fig 7B**). Together, our data indicate that the heightened resistance of TLR9^-/-^ mice to fatal *Ehrlichia* infection compared to MyD88^-/-^mice could be due to abrogation of MyD88 and IFN-I pathways; both of which clearly play synergistic pathogenic roles in induction of inflammasome activation and development of *Ehrlichia*-induced liver injury and toxic shock.

Our data demonstrate that MyD88 deficiency partially attenuates mRNA expression of IRF7 and IFNβ (**Fig 7A**), suggesting that MyD88 could mediate, in part, inflammasome activation by promoting IFN-I response. However, stimulation of IOE-infected MyD88^-/-^ BMM with IFNβ did not restore IL-1β secretion. We have examined IFNAR expression, as a possible mechanism that account for failure to restore IL-1β in IOE-infected MyD88^-/-^ BMM. We did not detect significant difference in IFNAR expression, at mRNA level, between IOE-infected WT and MyD88^-/-^ cells (**Fig 7C**). Although we have not examined whether MyD88 signaling influenced the IFNAR expression at the protein level, we believe this is unlikely scenario based on other studies showing that MyD88 is dispensable for upregulation of IFNAR expression and the induction of interferon stimulated genes. Indeed, IFN-I response is known to be promoted via number of complementary and/or redundant pathways during viral infections in mice and humans [59, 68, 69]. Based on these data, we conclude that failure to induce IL-1β in IOE-infected MyD88^-/-^ BMM is not due to attenuated expression of IFNAR, but possibly due to defective transcriptional upregulation of pro-IL-1β and pro-caspase-1/11 (signal 1) as described above.

Data from our previously published studies [8, 26] suggest that multiple inflammasome complexes including NLRP3, AIM2, and NLRC4 are upregulated during infection with virulent *Ehrlichia* and may play a role in the development of *Ehrlichia*-induced liver injury. The current study does not support a role for AIM2 in *Ehrlichia*-induced liver injury. Similarly, although expression of NLRC4 was downregulated in infected MyD88^-/-^ compared with WT mice, NLRC4 is less likely to be a critical inflammasome complex in our model since *Ehrlichia* does not posses flagella or type III secretion system effectors, which are known PAMPs for NLRC4 activation. Further studies are required to directly examine the contribution of NLRC4 or other inflammasome complexes in our model. Nevertheless, we believe that NLRP3 is one of the major inflammasome complex that contributes to the pathogenesis of *Ehrlichia*-induced liver injury and toxic shock. Our data demonstrating MyD88-dependent block of mitochondrial autophagy (mitophagy) suggest that oxidized mitochondrial DNA (mtDNA) or, ROS are potential DAMPs that induce activation of NLRP3 inflammasome. mtDNA can also provide positive feedback amplification of inflammasome pathways by triggering TLR9, NLRs, and cytosolic DNA sensors that induce type I IFN. This does not exclude the possibility that ehrlichial ligands (PAMPs) such as; bacterial DNA or type I and IV secreted effectors [70, 71], induces activation of the TLR9/MyD88 and inflammasome pathways, respectively.

**Fig 10:**
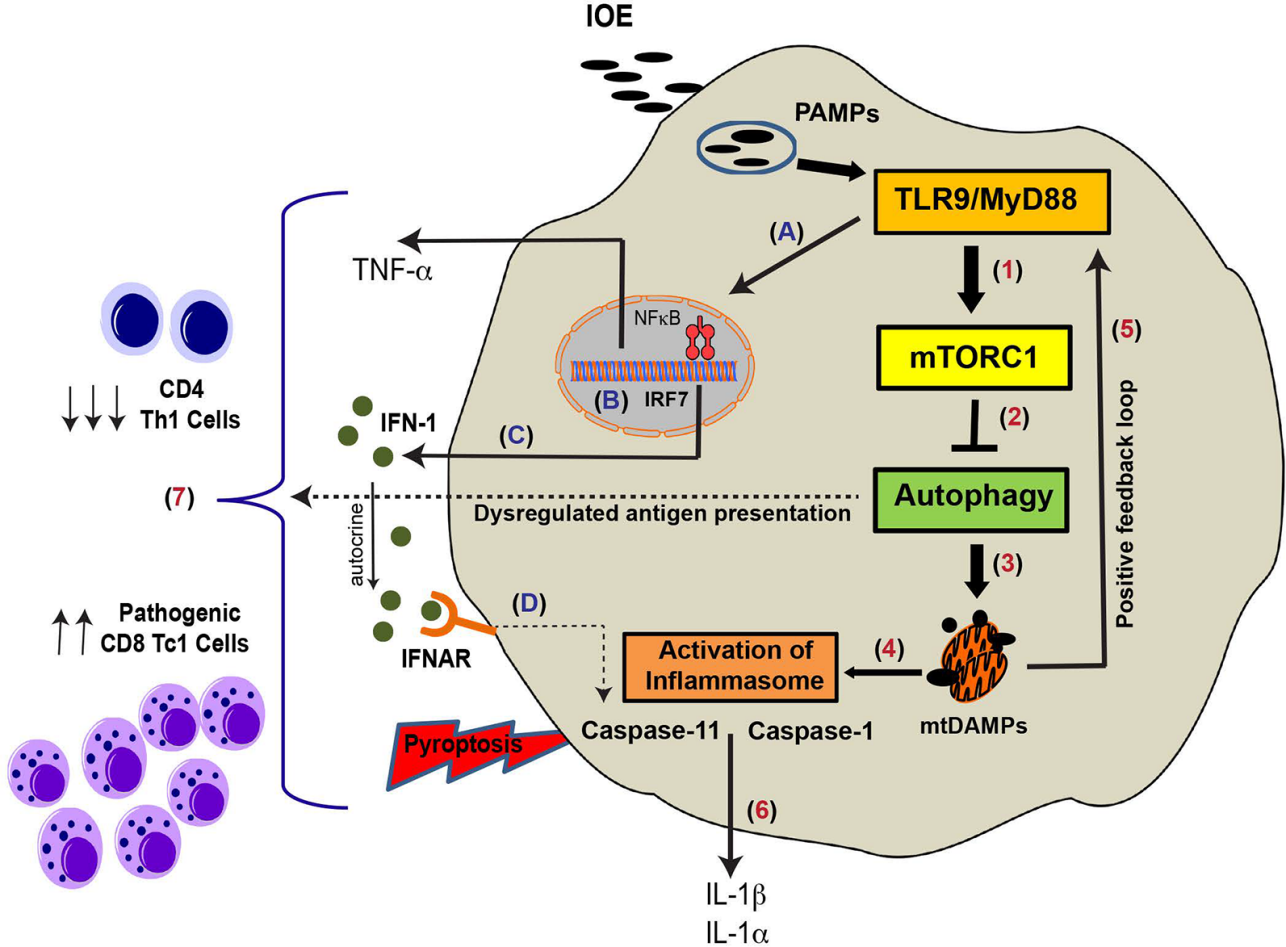
Schematic representation of MyD88-mediated inflammasome activation and immunopathology during fatal *Ehrlichia* infection. Infection of macrophages (main target cells) with virulent LPS-negative *Ehrlichia* triggers TLR9/MyD88 signaling and increased expression of pro-inflammatory cytokines (e.g.TNF-α) and upregulation of pro-IL-1β and inflammasome components (e.g. NLRP3), as well as induction of IRF7 and production of IFN-I (A-C). Recognition of PAMPs triggers TLR9 signaling and downstream MyD88 adaptor molecule activation, which in turn blocks autophagy induction in macrophages via mTORC1 activation. MyD88-induced block of autophagy can result in defective mitophagy and accumulation of mitochondrial DAMPs secondary to IOE-induced mitochondrial damage (1–4). These PAMPs and mitochondrial DAMPs can further induce TLR9 signaling, in a positive feedback loop (5), as well as activation of canonical (caspase 1 dependent) and non-canonical (caspase 11 dependent) inflammasome pathways (4). In addition, IFN-I produced by infected macrophages binds in an autocrine manner to IFNAR, which in turn induces activation of caspase 11 (D). This causes secretion of IL-1β/IL-1α and pyroptosis of infected macrophages (6). Defective autophagy can negatively influence MHC-II antigen presentation, which would lead to reduced proliferation of CD4^+^ Th1 cells (7). In addition, excessive inflammasome activation and host cell death promote expansion and activation of pathogenic CD8^+^ T cells (7), causing further liver damage.

In conclusion, based on our results, we propose a novel mechanism that explains the immunopathology and suppression of protective immunity during *Ehrlichia*-induced toxic shock **(Fig 10).** Sensing of vacuolar ehrlichial ligands (e.g. bacterial DNA) by TLR9 can trigger MyD88 signaling, which in turn causes activation of mTORC1 and suppression of autophagy. As *Ehrlichia* exploit autophagosomes to obtain nutrients and survive, MyD88-mediated mTORC1 activation and subsequent inhibition of autophagy induction is, therefore, a host-protective mechanism. On the other hand, MyD88-mediated partial block of autophagy flux/mitophagy contributes to excessive inflammation and immunopathology via activation of NF-κB and inflammasome (s). Importantly, TLR9/MyD88 signaling contributes to IFNβ/IFNαR- mediated non-canonical, caspase-11-dependent inflammasome activation and inflammatory host cell death. Cross presentation of infected apoptotic cells by dendritic cells and other antigen presenting cells (APCs), together with excessive inflammatory environment elicit activation of CD8^+^ T cells [72], which in turn cause further host cell death as suggested by our previous studies[3, 6, 23, 25]. In addition, IOE-induced inhibition of autophagy via MyD88 could impair MHC-class II mediated antigen presentation resulting in decreased expansion of Ag-specific CD4^+^ Th1 cells. Decreased magnitude of protective CD4^+^ Th1 response would result in uncontrolled bacterial replication that further induces excessive inflammation through a positive feedback loop. In-depth understanding of the intricate host-microbial interactions during infection with these obligate intracellular bacterial human pathogens will provide new insights for the development of effective therapeutics, diagnostics, as well as preventive strategies against fatal *Ehrlichia*-induced toxic shock.

## Material and methods

### Ethics statement

This study was conducted according to the principles species in the Declaration of Helsinki and under ethical Guidelines (University of Pittsburgh Institutional Review Board). All animal studies and procedures were carried out in strict accordance with the recommendations in the Guide for the Care and Use of Laboratory Animals of the National Institute of Health’s office of Laboratory Animal Welfare; the Assurance Number is A3187-01. The Division of Laboratory Animal Resources at University of Pittsburgh is accredited by the American Association for the Assessment and Accreditation of Lab Animal Care (AAALAC). The protocol (IACUC protocol number: 14125020) was approved by the Institutional Animal Care and Use Committees, University of Pittsburgh.

#### Mice and *Ehrlichia* infection

Female 7–8 week old C57BL/6 mice deficient in MyD88^-/-^, AIM2^-/-^, TLR9^-/-^, TLR7^-/-^ and wild type (WT) mice were obtained from Jackson Laboratory (Bar Harbor, ME), and were intraperitoneally (i.p.)-infected with a high lethal doses of IOE inoculum of 10^3^−10^4^ organisms/mouse. In certain experiments, mice were infected with IOE and treated with rapamycin (10mg/Kg) i.p. to enhance autophagy at early time points (day 1–3) following infection. Infected mice were monitored daily for signs of illness and survival. All mice were maintained in a specific pathogen-free environment at the University of Pittsburgh Animal Care Facility.

### Isolation and infection of Bone marrow derived macrophages (BMM)

Bone marrow was isolated from naïve, WT, MyD88^-/-^, TLR7^-/-^ and TLR9^-/-^ mice and prepared as described previously [73, 74]. Briefly, femurs were excised and flushed under aseptic conditions. Bone marrow cells were seeded in 100 mm petri dishes at 1x10^6^ cells/10ml/dish in DMEM/F12-GlutaMAX (Invitrogen) supplemented with 10% FBS (Invitrogen), 20ng/ml MCSF (PeproTech), 10 mM Hepes (Invitrogen), and 10 mM glutamine (Invitrogen). Approximately 4 ml of fresh media was added after 3 days of culture. On day 6, cells were collected and the BMM were characterized by flow cytometry and stained with fluorophore conjugated antibodies specific to CD11b, CD11c, and F4/80. The number of CD11c^-^CD11b^+^F4/80^+^ BMM isolated by this method was ~ 95%. BMM collected on day 6 were seeded into 12 well plates for 20 h prior to infection at a density of 10^6^ cells/well in DMEM/F12 (Invitrogen) supplemented with 5% FBS, 20 ng/ml MCSF, 10 mM HEPES. Cell-free IOE organisms were prepared from IOE-infected splenocytes as previously described [3]. IOE organisms were added to the BMM cultures at MOI of 5. Control cells were cultured with mock control Ag (antigens prepared from uninfected splenocyte). To confirm that host cell death did not account for differences between experimental groups, we measured cell viability at all time points (4, 8, 12, 24, and 48 h p.i.) using trypan blue staining.

### Pharmacologic activators and inhibitors and determination of intracellular bacterial number

For analysis of autophagy flux, bone marrow macrophages (5X 10^6^ cells/ml) were treated with autophagy inhibitor, bafilomycin A1 (100nM, InvivoGen) 4h before the termination of IOE infection at 24h. For blocking internalization of ehrlichiae, cells were pre-treated for 1h with a cell-permeable inhibitor of dynamin, Dynasore (30μM, Abcam). For blocking mTORC1 activation, cells were treated with mTORC1 inhibitor, rapamycin (10μM, InvivoGen). For stimulation of cells with type I IFN cytokines, IFNβ (Millipore, 500 IU/ml) was added to the cells along with IOE infection. For LPS/ATP positive controls, cells were treated with 200 ng/ml LPS for 18-24h followed by 30 min stimulation with 5mM ATP. For inhibition of caspase-1 and caspase-11 activation, cells were treated with caspase-1 (Ac-YVAD-cmk, in vivoGene) and caspase 11 (40 μM- Wedelolactone; CAS- 524-12-9, Santa Cruz) inhibitors for 2h. Both caspase-1 and caspase-11 inhibitors are used according to manufacturer's recommendation, and were shown to significantly inhibit caspase- 1 and caspase-11 expression in cultured cells, respectively. However, these inhibitors may also non-specifically inhibit other caspases or apoptosis-related proteins. Infected culture without the inhibitors and uninfected cells were used as positive and negative controls for all the above experimental conditions, respectively. For detection of intracellular bacteria, cells were collected at 12h and 24h post-infection, and washed two times with PBS to remove the extracellular bacteria. The number of ehrlichiae was determined by qPCR or RT-PCR analysis of the *Ehrlichia* dsb gene or 16S rRNA, respectively, as described below. Supernatants were collected and stored at −80°C for cytokine analysis at a later time.

### *In vitro* stimulation and flow cytometry

Splenocytes from naïve or infected mice were stimulated *in vitro* with IOE antigens for 12h to determine the frequency of antigen-specific T cells. *In vitro* stimulated spleen cells were re-suspended in fluorescence-activated cell sorter-staining buffer at a concentration of 10^6^ cells/well. FcRs were blocked with a mAb (clone 2.4G2) against mouse cell surface antigens CD16 and CD32 for 15 min. The following fluorescence-conjugated Abs were used (all antibodies were purchased from either BD Biosciences or Biolegend): anti-CD3 (clone 145- 2C11), anti-CD4 (clone RM4-4), anti-CD8a (clone 53–6.7), anti-CD69 (clone RA3-6B2), anti-IL-10 (clone JES5-16E3), anti–TNF-α (clone MP6-XT22), anti–IFN-γ (clone XMG102). Isotype control mAbs, including FITC-, PE-, Percep, and allophycocyanin-conjugated hamster IgG1 (A19-3), rat IgG1 (R3-34), rat IgG2α (R35-95), mouse IgG2α (X39), mouse IgG2b (MPC-11), mouse IgG1 (X40), and rat IgG2b (A95-1). For intracellular cytokine staining, the splenocytes were stimulated with IOE antigen, with addition of BD Golgi Plug. Following blocking and primary antibody staining, cells were permeabilized with CytoFix-CytoPerm kit (BD Biosciences) for detection of IFN-γ and IL-10. Lymphocyte and granulocyte populations were gated based on forward and side-scatter parameters. Approximately 50,000 events were collected for the spleen cells and 100,000 for the LMNCs using the BD-LSR II (BD Immunocytometry Systems, San Jose, CA) flow cytometry, and the data were analyzed using FlowJo software (TreeStar, Ashland, OR).

### Western blot analysis

Liver tissues and BMM were lysed in T-PER lysis buffer or RIPA buffer (Thermo Fisher Scientific, Waltham, MA) respectively, supplemented with protease inhibitors and 1 mM phenylmethylsulphonyl fluoride (PMSF). Protein extraction was performed at 4°C for 30 min and the protein content of the lysates was measured using a Bicinchoninic Acid Assay Kit (Pierce). Lysates (15–40 ug) were resolved in 4% to 20% gradient SDS–PAGE under reducing conditions. After electrophoresis, proteins were transferred onto PVDF membranes (BioRad), and blocked for 1 h in Tris-buffered saline (TBS) containing 5% non-fat milk and 0.1% Tween 20. Blots were probed with the appropriate primary antibodies and peroxidase-conjugated bovine anti-rabbit secondary antibodies (1:10000) (Santa Cruz Biotechnology). The membranes were processed and probed with the following antibodies, according to standard protocols: anti–caspase-1 (1:100) (EMD Millipore, Billerica, MA), anti–caspase-11 (1:100) and anti-LC3B (Sigma-Aldrich, St. Louis, MO) and anti-IL-1β (1:100, GeneTex). The following antibodies were from Cell Signaling Technology, Inc., and used at 1:1000 dilution; anti-beclin 1, anti-p62 (SQSTMI), anti-phospho 4E-BP1, anti-AkT, anti-phosphoAkT, anti-S6, anti-phospho S6, anti-Atg5. Specific signals were developed using the ECL-Plus system (GE). Blots were stripped with Restore Western Blot Stripping Buffer (Pierce) and re-probed with anti-GAPDH (Sigma, 1:5000) or anti-beta actin (Abcam; 1:2500) as loading controls. The density of bands in Western blots was determined using ImageJ software version 1.48 (NIH, Bethesda, MD). The ratio of LC3II:I was determined by normalization of the LC3II and LC3I to GAPDH, and then presented the normalized LC3II:LC3I ratio.

### Immunofluorescence staining and confocal microscopy

Staining of LC3 puncta, p62 aggregates, acidified lysosomes, and mitochondria (healthy and damaged) and quantification by confocal microscopy were performed as previously described [35, 75]. Briefly, BMM cultured on cover slips were infected with IOE at MOI of 5. Negative and positive controls included are uninfected cells (stimulated with mock Ags) or LPS stimulated cells, respectively. Cells were then washed 3X with PBS, fixed with 2% paraformaldehyde for 20 min, and permeabilized with 0.1% Triton X-100 in PBS for 30 min. After blocking with 5% BSA (Sigma-Aldrich, A2153) for 60 min, the primary antibodies; anti-LC3 (Sigma, 50 ug/mL), anti-p62 (Cell Signaling, 1:100) were added for 1 h at room temperature. Cells were washed and then incubated with fluorescent labeled anti-rabbit secondary antibody DyLight (VectaFluor, 1:500) for 1 h. Nuclei were stained with DAPI and cells were analyzed by confocal microscopy (Olympus Flouview 1000). For localization of IOE with LC3, infected cells and uninfected controls were incubated with polyclonal rabbit anti-*Ehrlichia chaffeensis* antibody (added at 1: 1000 dilution) that cross-react with IOE organisms as we described before [3, 6] for 2h followed by incubation with fluorescent labeled anti-rabbit secondary antibodies. Analysis of mitochondria (healthy and damaged) or acidified lysosome was performed using Mitotracker Red (#M-7512, Life Technologies) or LysoTracker Red (#L-12492, Thermofisher), respectively, at 37°C for 1 h and assessed with a confocal microscope (Olympus flouview 1000).

### Image analysis

Analysis of LC3 puncta or colocalization was performed using the NIH Image J software package and analyzing 40–50 cells per group from 3 independent experiments. The LC3 puncta was identified as highly fluorescent green aggregates. Colocalization analysis was performed with a Olympus fluorescent microscope equipped with a CCD camera and magnifier software, which allows capturing images digitally. The colocalization images is analyzed using PCI software from a Olympus fluorescent microscope by counting number of yellow aggregate per cell, with 30–45 cells per experiment and data collected from 3 independent experiments. Results were presented as average LC3 puncta per cell, percentage of cells with more than 5 LC3 puncta/field, or the number of colocalized puncta/cell. At least 10 fields of view for each sample were quantified for each experimental group across at least three independent experiments Data were analyzed as average ± standard deviation.

### JC-1 mitochondrial membrane potential assay

Mitochondrial membrane potential was assayed using JC-1 (Biotium, Fremont, CA) kit according to the manufacturer’s protocol. Briefly 100μl of the JC-1 staining solution was added per ml culture medium of coverslip cultured naïve and IOE infected WT and MyD88^-/-^ BMM (24h p.i.), and incubated for 30 minutes at 37°C. Photographic images were taken using an Olympus confocal microscope and quantification performed using Image J as described above.

### Transmission electron microscopy (TEM)

Bone marrow derived macrophages from WT or MyD88^-/-^ mice were infected with IOE at MOI of 5, in the presence or absence of rapamycin or Dynasore for 24h. After infection and treatment, cells were fixed with 2.5% glutaraldehyde 0.1 M phosphate buffer (pH 7.4), and post-fix monolayer in 1% osmium tetroxide with 1% potassium ferricyanide, and dehydrated with a graded series of alcohol. Invert beam capsules of resin over relevant areas of monolayers and polymerize. Ultrathin sections were processed by transmission electron microscopy (JEM-1011 Transmission Electron Microscope, JEOL Ltd.) For quantitative evaluation of autophagosomes and mitochondria in BMM, 10 image fields (10,000 X) were selected for each sample.

### Bacterial burden analysis by quantitative real-time PCR (qPCR) and immunofluorescence

Total DNA was isolated from liver tissues (*in vivo*) or bone marrow macrophages (*in vitro*) using the DNeasy Blood and Tissue kit (QIAGEN). Bacterial burden was determined by quantitative Real-Time PCR, using an ABI 7500 FAST System (Applied Biosystems, USA) and using specific primer sets (S1 Table) amplifying the IOE dsb gene as previously described [76]. The eukaryotic housekeeping gene *gapdh* was amplified using the GAPDH primers (S1 **Table**). The absolute IOE *dsb* copy number was determined using a standard curve and was normalized to qPCR-detected levels of the *gapdh* in the same sample and expressed as copy number per 10^4^ copies of *gapdh*. The number of intracellular *Ehrlichia* within BMM was measured at early stage of infection by immunofluorescence microscopy since qPCR cannot distinguish between live and dead bacteria.

In certain experiment, the *Ehrlichia* copy number determined by *dsb* was correlated with the copy number based on detection of *Ehrlichia* 16S rRNA in the samples, which measures viable bacteria as described before [76–78]. Briefly, mRNA was isolated from the samples and quantitative RT-PCR was performed as described below.

### RNA extraction and quantitative RT-PCR (qRT-PCR)

RNA from the liver tissues or bone marrow macrophages was extracted using TRIzol Reagent (Invitrogen Life Technologies, Carlsbad, California, USA). RNA (2 μg) was reverse transcribed in a final volume of 20 μl using RT^2^ first strand kit (Qiagen, USA), as specified by the manufacturer, and then subjected to PCR using specific primer sets (S1 Table). qRT-PCR was conducted using an ABI 7500 FAST System (Applied Biosystems, USA). cDNA (100 ng) was subjected to qRT-PCR in a 25 μl reaction volume using SYBR-Premix (Qiagen). The GAPDH gene was amplified for normalization of the cDNA amount used in qRT-PCR. Reactions were carried out in triplicate, and the data were analyzed using the 2^−ΔΔCt^ method.

### Histopathology and terminal deoxynucleotidyl transferase dUTP nick end labeling (TUNEL) assay

Liver samples were fixed in a 10% solution of neutral buffered formalin, dehydrated in graded alcohols, embedded in paraffin wax, and stained with hematoxylin and eosin (H&E). Histological sections were evaluated qualitatively for morphological differences and quantitatively for apoptosis (by TUNEL immunohistochemical assays). TUNEL staining (Research Histology Services, University of Pittsburgh) was performed on the formalin-fixed, paraffin-embedded tissue sections. Apoptotic Kupffer cells and hepatocytes were counted in 10 high-power fields (HPFs) for each mouse. Stained slides were viewed under an Olympus (Tokyo, Japan) BX40 microscope and were scanned using a Mirax MIDI slide scanner (Carl Zeiss Microscopy, Jena, Germany); images were captured using Pannoramic Viewer software (3DHistech, Budapest, Hungary).

### LDH release assay

Cell culture supernatants and cell pellets from infected or uninfected bone marrow-derived macrophages (BMM) isolated from WT mice were collected at 24h p.i. and assayed for LDH activity using the CytoToxo96 LDH-release kit (Promega) following the manufacturer’s recommendations.

### ELISA

The levels of IL-1α, IL-1β, IL-10, and TNF-α in serum of infected mice or produced by the WT and MyD88^-/-^ BMM, IOE infected and uninfected culture supernatants were determined by commercially available enzyme-linked immunosorbent assay (ELISA(eBioscience, San Diego, CA; Vienna, Austria)) kits according to the manufacturer’s instructions.

### Statistical analysis

All of the data presented are representative of at least three independent experiments that yielded similar results. Two group analyses was performed using an unpaired two-tailed *t*-test. For comparison of multiple experimental groups, we used one-way analysis of variance (ANOVA) with Bonferroni’s procedure. To determine whether the difference in survival between different mice groups was significant, data were analyzed by the Breslow-Wilcoxon Test. All statistical analyses were performed using Graph Pad Prism (GraphPad Software Inc., La Jolla, CA, USA). Data are represented by means and standard deviations (SD). Differences with *P* values of <0.05, <0.01, and <0.001 were considered slightly (*), moderately (**), and highly (***) significant, respectively.

## Supporting information

S1 Table Primers used for amplification of host genes as well as quantification of *Ehrlichia* in the *in vivo* and *in vitro* experiments. (DOCX)

S1 Fig MyD88 signaling plays a role in induction of protective adaptive immune responses against *Ehrlichia*. Spleens were harvested from the naïve or infected WT and MyD88^-/-^ mice on day 7 p.i., and splenocytes were stimulated *in vitro* with IOE antigens (Ags) (A) Representative gating strategy to define splenic CD4 and CD8 T cells by flow cytometry. (B) Expression of activation marker CD69 and the intracellular expression of IFNγ. (C) The percentage of IL-10 producing CD4 and CD8 T cells in naïve WT, IOE-infected WT, and IOE-infected MyD88^-/-^ mice determined by flow cytometry. (D) Absolute number of CD4 and CD8 T cells in the spleen of indicated mice groups, which was calculated by multiplying the percentage of each cell subset by the total number of splenocytes from each mouse. (E) Ratio of IL-10: IFNγ-producing CD4+ T cells in the spleen of uninfected and infected WT and MyD88^-/-^ mice on day 7 p.i. (F) Ratio of IL-10: TNF-α in mice sera at day 7 p.i. Data shown representative of three mice per group and of three independent experiments. Results presented as the mean ± SD (* P<0.05). *P*-values calculated by Student’s *t*-test, paired are indicated above the graph.
(TIF)

S2 Fig AIM2 inflammasome is not required for caspase-1-mediated inflammasome activation and liver injury in response during infection with virulent *Ehrlichia*. (A) Survival of naïve (uninfected) and IOE-infected WT and AIM2^-/-^ mice, showing 100% mortality of AIM2^-/-^ at 8–10 days p.i. similar to infected WT mice (n = 9/group). (B) H&E (upper) and TUNEL (lower) staining of liver sections from AIM2^-/-^ and WT mice on day 7 p.i. (C) Quantification of TUNEL positive Kupffer cells and hepatocytes per high power field (hpf) in AIM2^-/-^ and WT mice. (D) Levels of IL-1β in the sera of naïve and IOE-infected WT and AIM2^-/-^ mice at day 7 p.i. (E) Representative Immunoblots of active/cleaved caspase-1 and IL-1β in liver lysates from uninfected and IOE-infected WT mice, compared with AIM2^-/-^ mice on day 7 p.i. β-actin used as loading control. Data shown as mean ±SD from three independent experiments with 3 mice/group. ns = not significant.
(TIF)

S3 Fig Kinetic analysis of autophagy induction in IOE infected WT and MyD88^-/-^ BMM. (A) Confocal immunofluorescence staining of LC3 punctae (green) in uninfected and IOE-infected BMM from WT and MyD88^-/-^ mice at 0, 4, 8, 12 & 24h p.i. DAPI nuclear stain is blue (B) Quantification of percentage of cells with more than 5 LC3 puncta by confocal microscopy. All results are presented as mean ± SD from three different experiments (* P<0.05).
(TIF)

S4 Fig Virulent *Ehrlichia* exploit MyD88 to inhibit autophagosomal-lysosomal fusion. (A) Confocal immunofluorescence colocalization (yellow) of LC3 (green) and lysosomes (LysoTracker; red) in uninfected and IOE-infected BMM from WT and MyD88^-/-^ mice at 12h p.i. LPS (200ng/mL) was used as a positive control. (B) Percentage of Lysosome colocalization with LC3 per cell was quantified with the use of ImageJ software. Values are (mean ± SEM) from three different experiments. (* P<0.05).
(TIF)

S5 Fig Defective co-localization of *Ehrlichia* with the Lysosomes. (A) Confocal immunofluorescence staining of IOE (polyclonal rabbit anti-*Ehrlichia chaffeensis* antibody; green) and lysosomes (LysoTracker; red) in uninfected and IOE-infected BMM from WT and MyD88^-/-^ mice at 24h p.i. Data show no colocalization of IOE with lysosomes in IOE-infected WT and MyD88^-/-^ BMM.
(TIF)
