# Supplementary material for: MyD88-dependent inflammasome activation and autophagy inhibition contributes to Ehrlichia-induced liver injury and toxic shock
Source: PLoS Pathog. 2017 Oct 19;13(10):e1006644. doi: 10.1371/journal.ppat.1006644 (PMC5663626; doi:10.1371/journal.ppat.1006644)
Supplement: S1 Table — (DOCX) [file ppat.1006644.s001.docx]

| **Primers from Integrated DNA Technologies (IDT)** | | |
| --- | --- | --- |
|  |  |  |
| **Primer Name** | **Forward 5' -- 3'** | **Reverse 5' -- 3'** |
| **DSB** | **CAG GAT GGT AAA GTA CGT GTG A** | **TAG CTA ACG CTG CCT GAA CA** |
| **GAPDH** | **CAA CTA CAT GGT CTA CAT GTT C** | **TCG CTC CTG GAA GAT G** |
| **IL-1β** | **ACC CTG CAG TGG TTC GAG** | **TTG CAC AAG GAA GCT TGG** |
| **NLRP3** | **CGA GAC CTC TGG GAA AAA GCT** | **GCA TAC CAT AGA GGA ATG TGA TGT ACA** |
| **NLRC4** | **GCA ATG GTT TAT CAG CAC G** | **TGC CAC ACT CTA CGA AGG A** |
| **AIM2** | **AGG CAG TGG GAA CAA GAC AG** | **AAA CTT CCT GAC GCC ACC C** |
| **Caspase-1** | **ATC ATT TCC GCG GTT GAA T** | **AAT TGC TGT GTG CGC ATG T** |
| **Caspase-11** | **ACA ATG CTG AAC GCA GTG AC** | **CTG GTT CCT CCA TTT CCA GA** |
| **ATG5** | **AAAGATGTGCTTCGAGATGTGT** | **CACTTTGTCAGTTACCAACGTCA** |
| **IFNAR2** | **GGA TGG CAG TGA CAG TGAC** | **ATG GAG AAC CCT CAG AAA CAC** |
| **16S rRNA** | **AGC AAT GCC TCC TGC ACC ACC AAC** | **CCA CAT CAC CCC TCT ACC TC** |
| **RT^2^ qPCR Primers from Qiagen** | | |
|  |  |  |
| **Primer Name** | **Catalog #** | **Reference Position** |
| **GAPDH** | **PPM02946E** | **309** |
| **IFNβ-1** | **PPM03594C** | **345** |
| **IRF7** | **PPM04696E** | **1414** |
