# Supplementary figures and images for: MyD88-dependent inflammasome activation and autophagy inhibition contributes to Ehrlichia-induced liver injury and toxic shock
Source: PLoS Pathog. 2017 Oct 19;13(10):e1006644. doi: 10.1371/journal.ppat.1006644 (PMC5663626; doi:10.1371/journal.ppat.1006644)

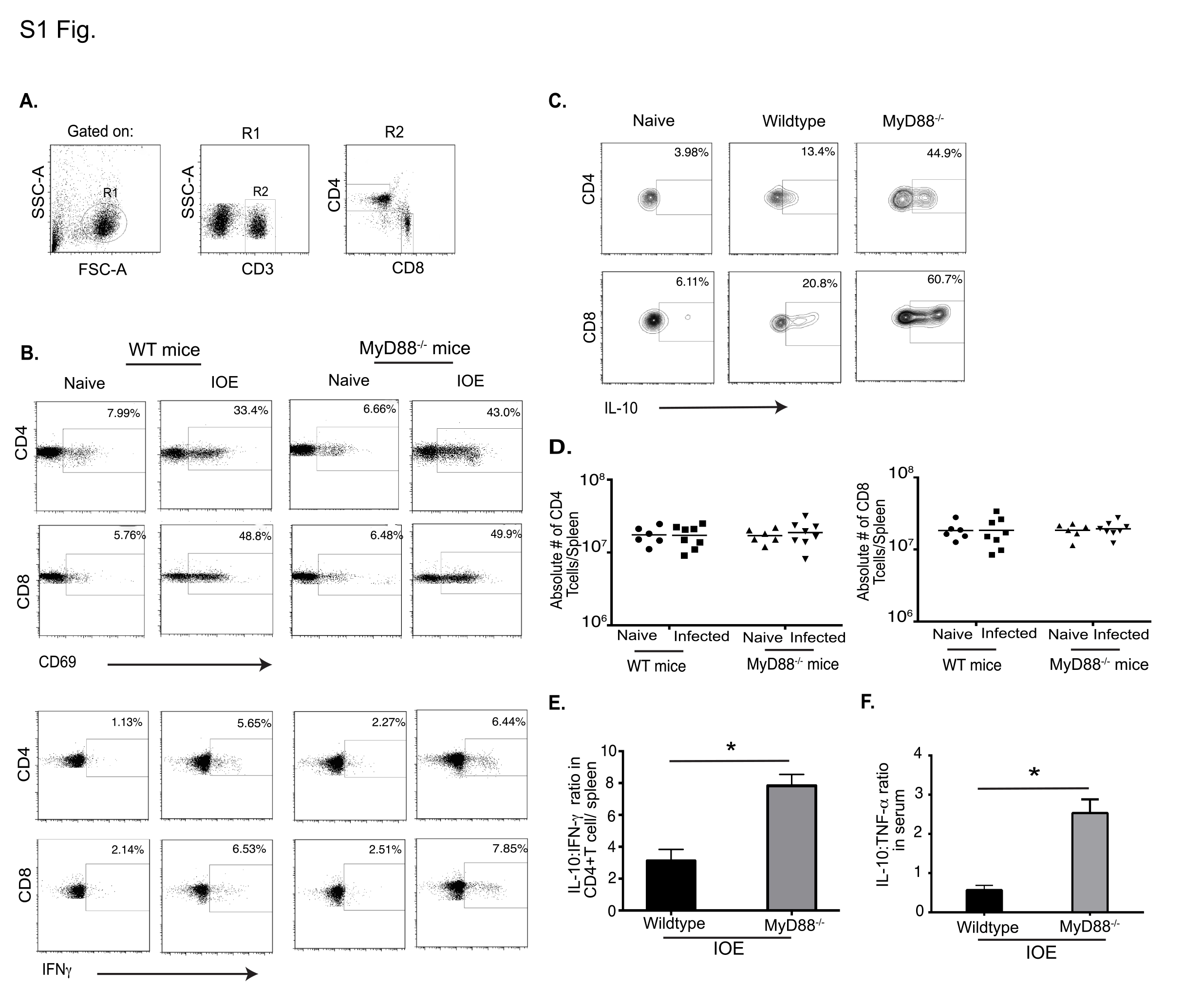

Supplement: S1 Fig — Spleens were harvested from the naïve or infected WT and MyD88-/- mice on day 7 p.i., and splenocytes were stimulated in vitro with IOE antigens (Ags) (A) Representative gating strategy to define splenic CD4 and CD8 T cells by flow cytometry. (B) Expression of activation marker CD69 and the intracellular expression of IFNγ. (C) The percentage of IL-10 producing CD4 and CD8 T cells in naïve WT, IOE-infected WT, and IOE-infected MyD88-/- mice determined by flow cytometry. (D) Absolute number of CD4 and CD8 T cells in the spleen of indicated mice groups, which was calculated by multiplying the percentage of each cell subset by the total number of splenocytes from each mouse. (E) Ratio of IL-10: IFNγ-producing CD4+ T cells in the spleen of uninfected and infected WT and MyD88-/- mice on day 7 p.i. (F) Ratio of IL-10: TNF-α in mice sera at day 7 p.i. Data shown representative of three mice per group and of three independent experiments. Results presented as the mean ± SD (* P<0.05). P-values calculated by Student’s t-test, paired are indicated above the graph. (TIF) [file ppat.1006644.s002.tif]

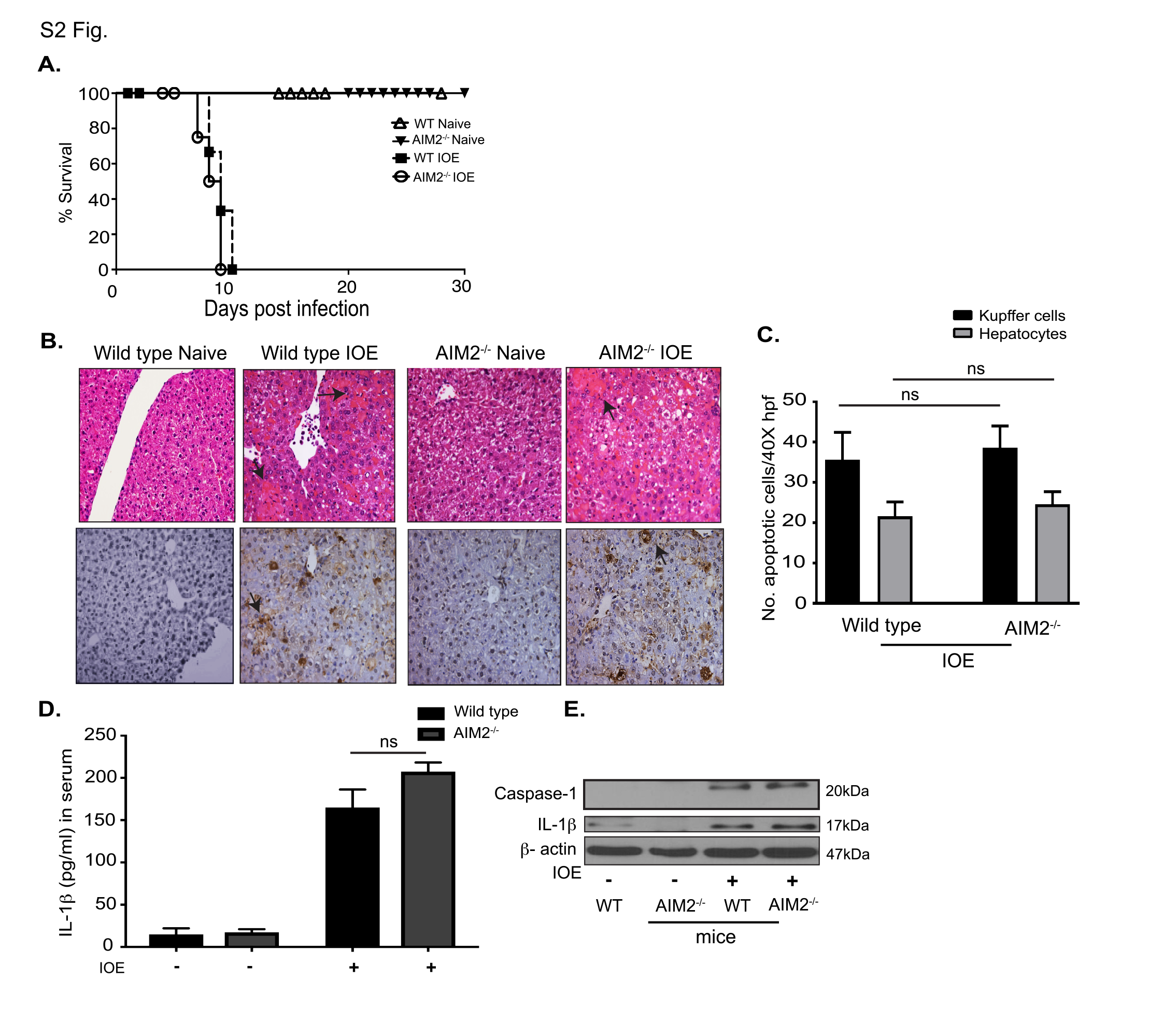

Supplement: S2 Fig — (A) Survival of naïve (uninfected) and IOE-infected WT and AIM2-/- mice, showing 100% mortality of AIM2-/- at 8–10 days p.i. similar to infected WT mice (n = 9/group). (B) H&E (upper) and TUNEL (lower) staining of liver sections from AIM2-/- and WT mice on day 7 p.i. (C) Quantification of TUNEL positive Kupffer cells and hepatocytes per high power field (hpf) in AIM2-/- and WT mice. (D) Levels of IL-1β in the sera of naïve and IOE-infected WT and AIM2-/- mice at day 7 p.i. (E) Representative Immunoblots of active/cleaved caspase-1 and IL-1β in liver lysates from uninfected and IOE-infected WT mice, compared with AIM2-/- mice on day 7 p.i. β-actin used as loading control. Data shown as mean ±SD from three independent experiments with 3 mice/group. ns = not significant. (TIF) [file ppat.1006644.s003.tif]

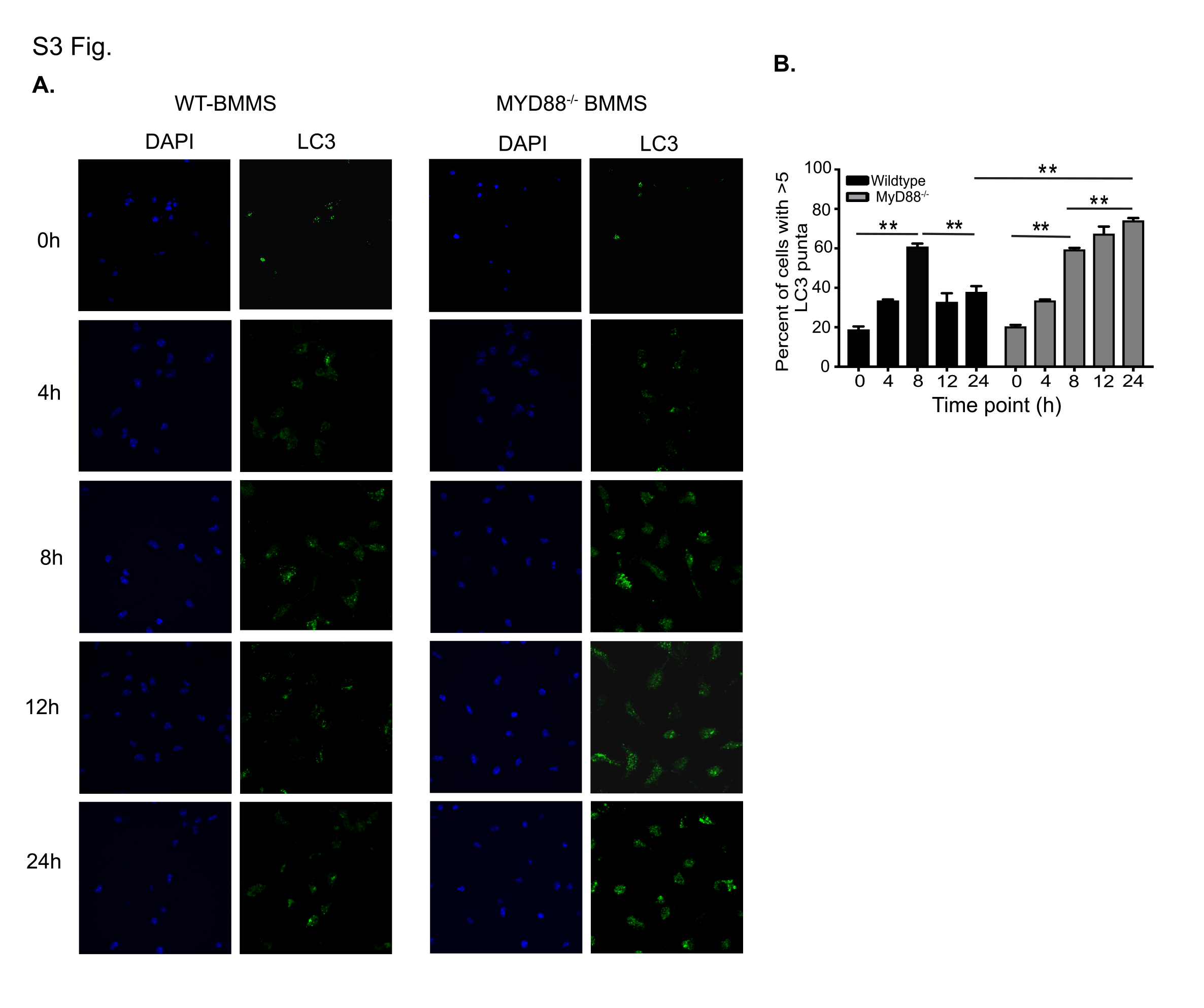

Supplement: S3 Fig — (A) Confocal immunofluorescence staining of LC3 punctae (green) in uninfected and IOE-infected BMM from WT and MyD88-/- mice at 0, 4, 8, 12 & 24h p.i. DAPI nuclear stain is blue (B) Quantification of percentage of cells with more than 5 LC3 puncta by confocal microscopy. All results are presented as mean ± SD from three different experiments (* P<0.05). (TIF) [file ppat.1006644.s004.tif]

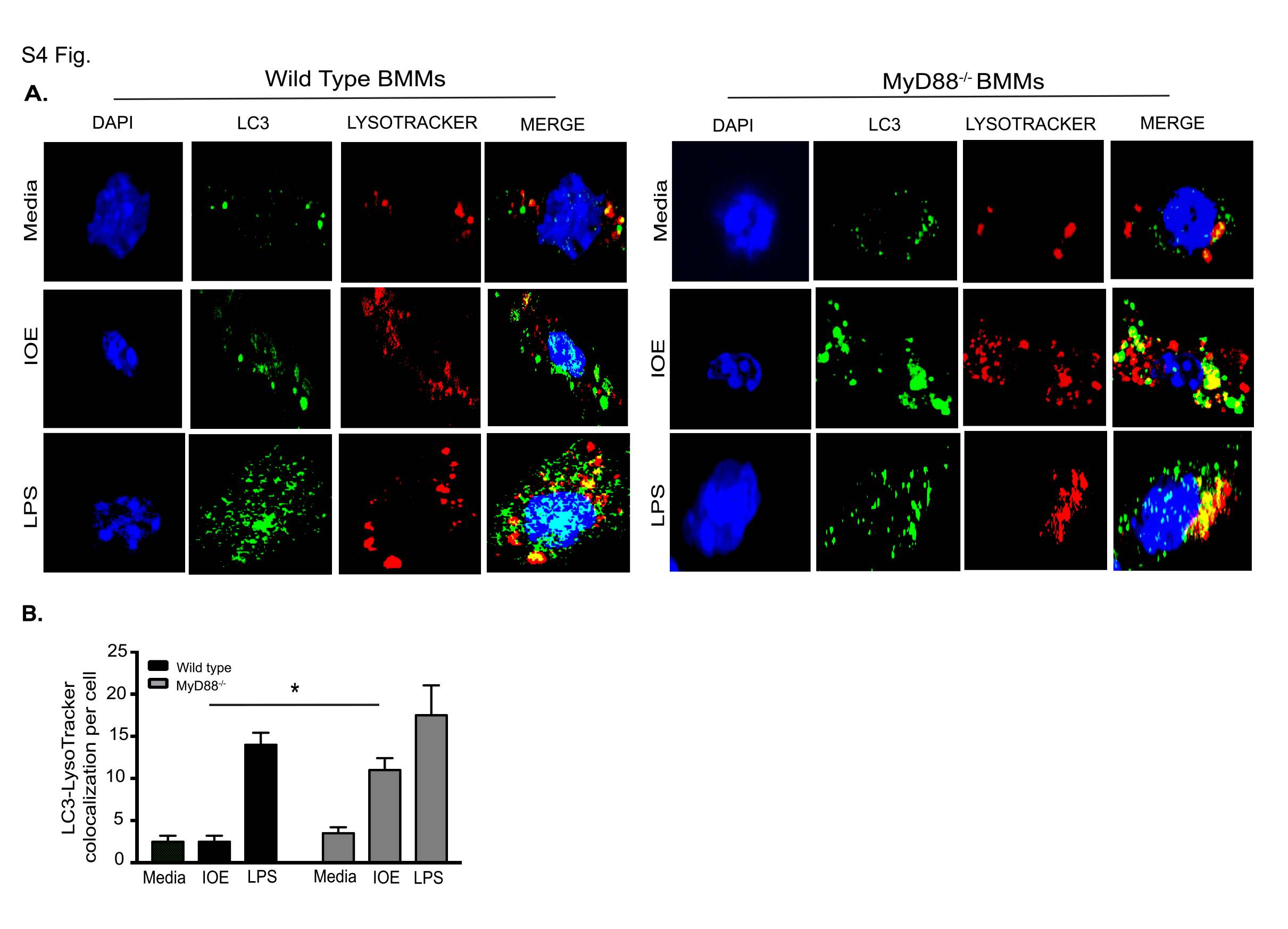

Supplement: S4 Fig — (A) Confocal immunofluorescence colocalization (yellow) of LC3 (green) and lysosomes (LysoTracker; red) in uninfected and IOE-infected BMM from WT and MyD88-/- mice at 12h p.i. LPS (200ng/mL) was used as a positive control. (B) Percentage of Lysosome colocalization with LC3 per cell was quantified with the use of ImageJ software. Values are (mean ± SEM) from three different experiments. (* P<0.05). (TIF) [file ppat.1006644.s005.tif]

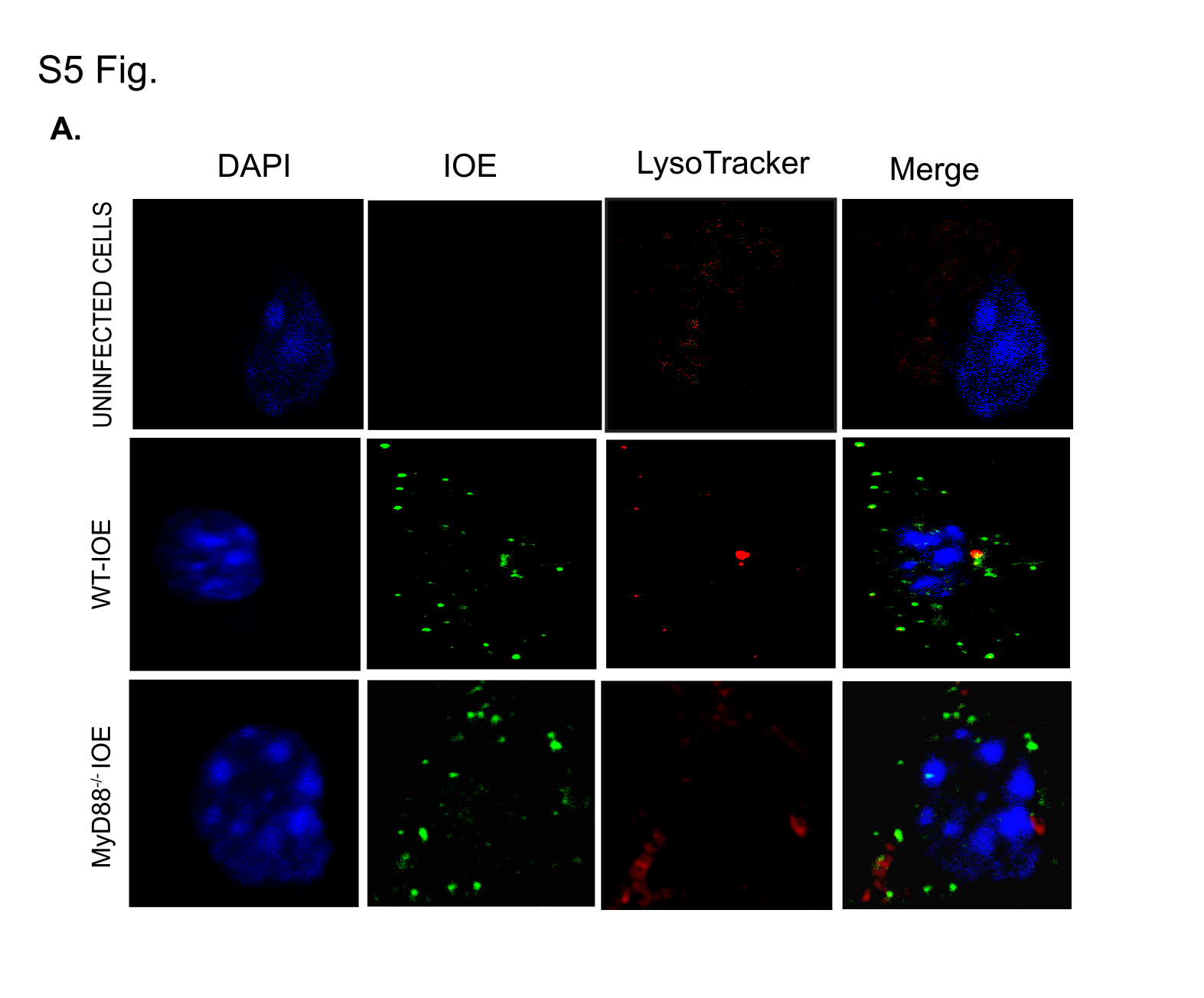

Supplement: S5 Fig — (A) Confocal immunofluorescence staining of IOE (polyclonal rabbit anti-Ehrlichia chaffeensis antibody; green) and lysosomes (LysoTracker; red) in uninfected and IOE-infected BMM from WT and MyD88-/- mice at 24h p.i. Data show no colocalization of IOE with lysosomes in IOE-infected WT and MyD88-/- BMM. (TIF) [file ppat.1006644.s006.tif]
